# Supplementary figures and images for: Multi-drug resistant (MDR) Gram-negative pathogenic bacteria isolated from poultry in the Noakhali region of Bangladesh
Source: PLoS One. 2024 Aug 1;19(8):e0292638. doi: 10.1371/journal.pone.0292638 (PMC11293736; doi:10.1371/journal.pone.0292638)

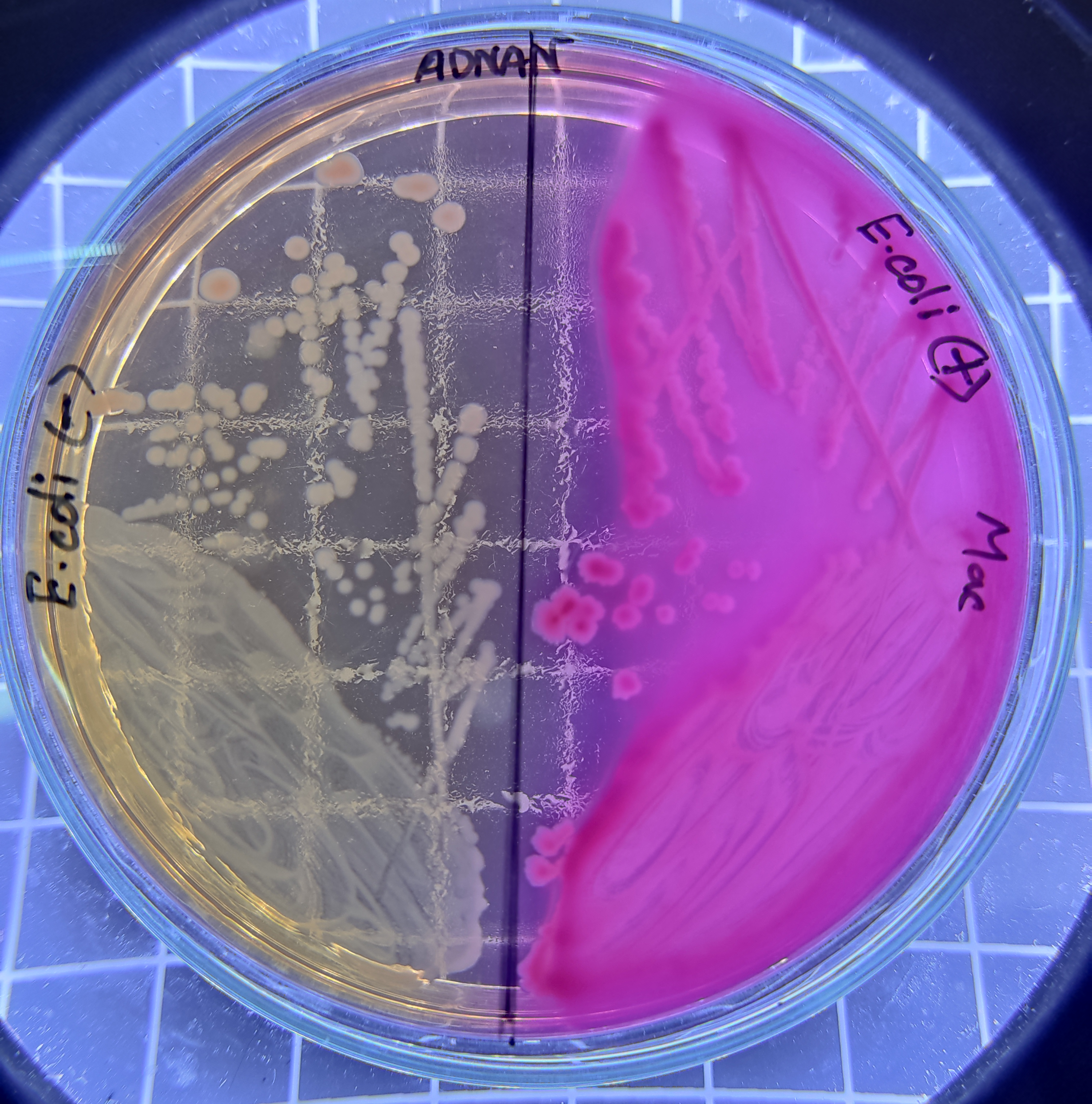

Supplement: S1 Fig — ((+) = Lactose Positive, (-) = Lactose negative Mac = MacConkey agar). (TIFF) [file pone.0292638.s001.tiff]

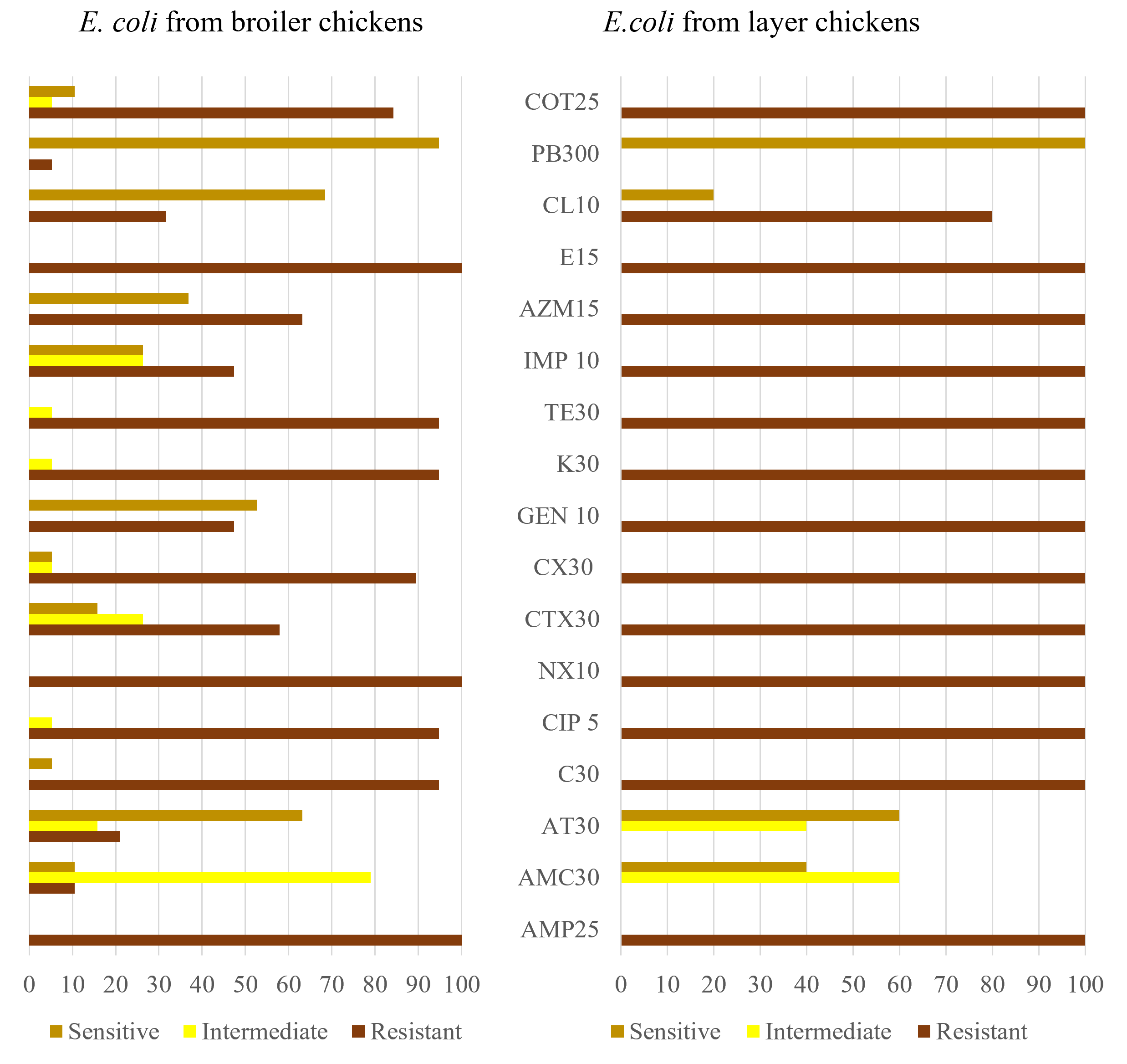

Supplement: S2 Fig — (Ampicillin (AMP25), Amoxicillin-clavulanic acid (AMC 30), Cefotaxime (CTX 30) Cefoxitin (CX 30) Ciprofloxacin (CIP 5) and Norfloxacin (NX 10), Aztreonam (AT 30), Gentamicin (GEN 10), Kanamycin (K 30), Azithromycin (AZM 30) Erythromycin (E 10), Imipenem (IMP 10), Chloramphenicol (C 30), Trimethoprim-Sulfamethoxazole (COT 25), Tetracycline (TE 30), Colistin (CL 10) and Polymyxin B (PB 300)). (TIF) [file pone.0292638.s002.tif]

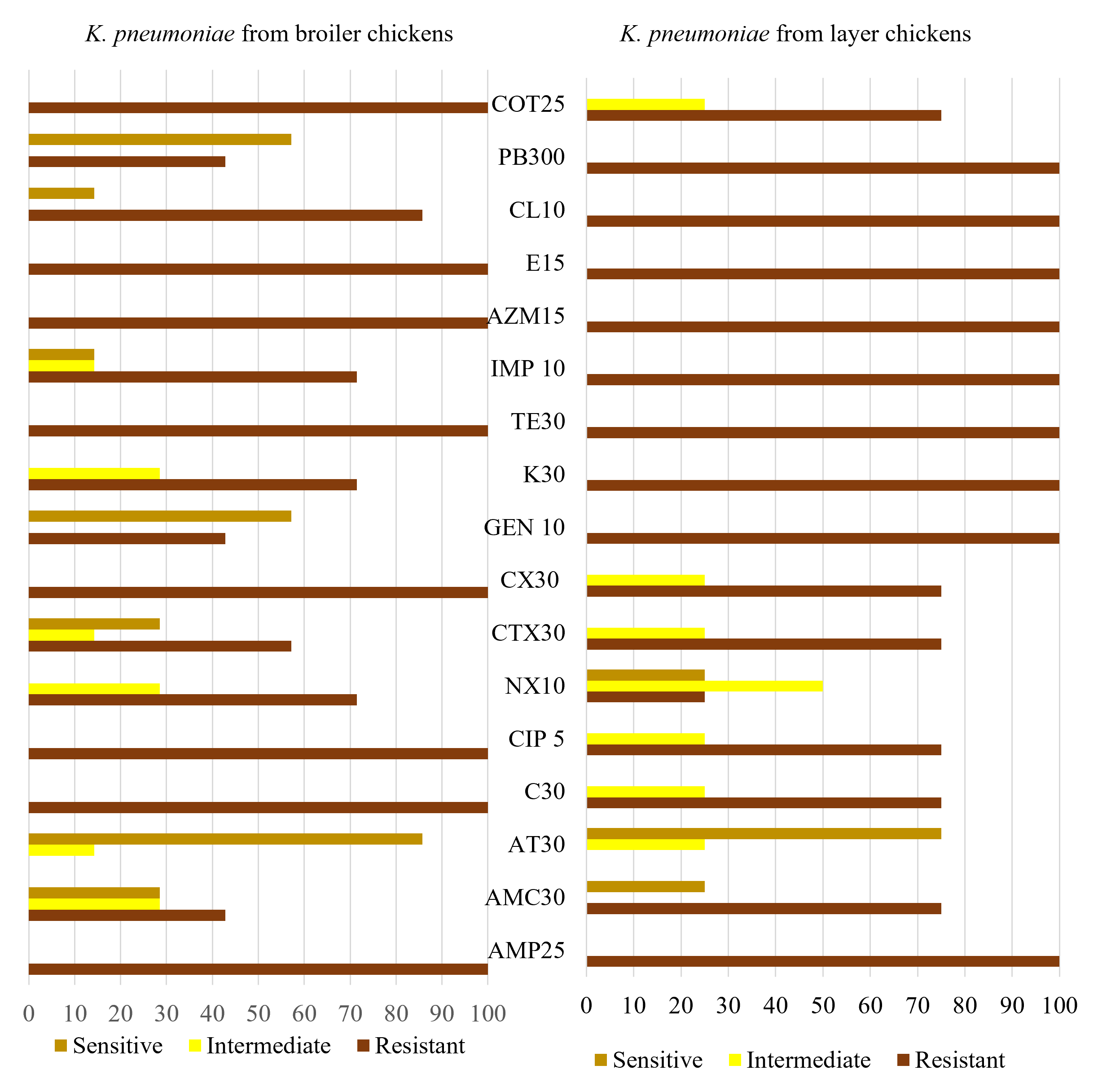

Supplement: S3 Fig — (Ampicillin (AMP25), Amoxicillin-clavulanic acid (AMC 30), Cefotaxime (CTX 30) Cefoxitin (CX 30) Ciprofloxacin (CIP 5) and Norfloxacin (NX 10), Aztreonam (AT 30), Gentamicin (GEN 10), Kanamycin (K 30), Azithromycin (AZM 30) Erythromycin (E 10), Imipenem (IMP 10), Chloramphenicol (C 30), Trimethoprim-Sulfamethoxazole (COT 25), Tetracycline (TE 30), Colistin (CL 10) and Polymyxin B (PB 300)). (TIF) [file pone.0292638.s003.tif]

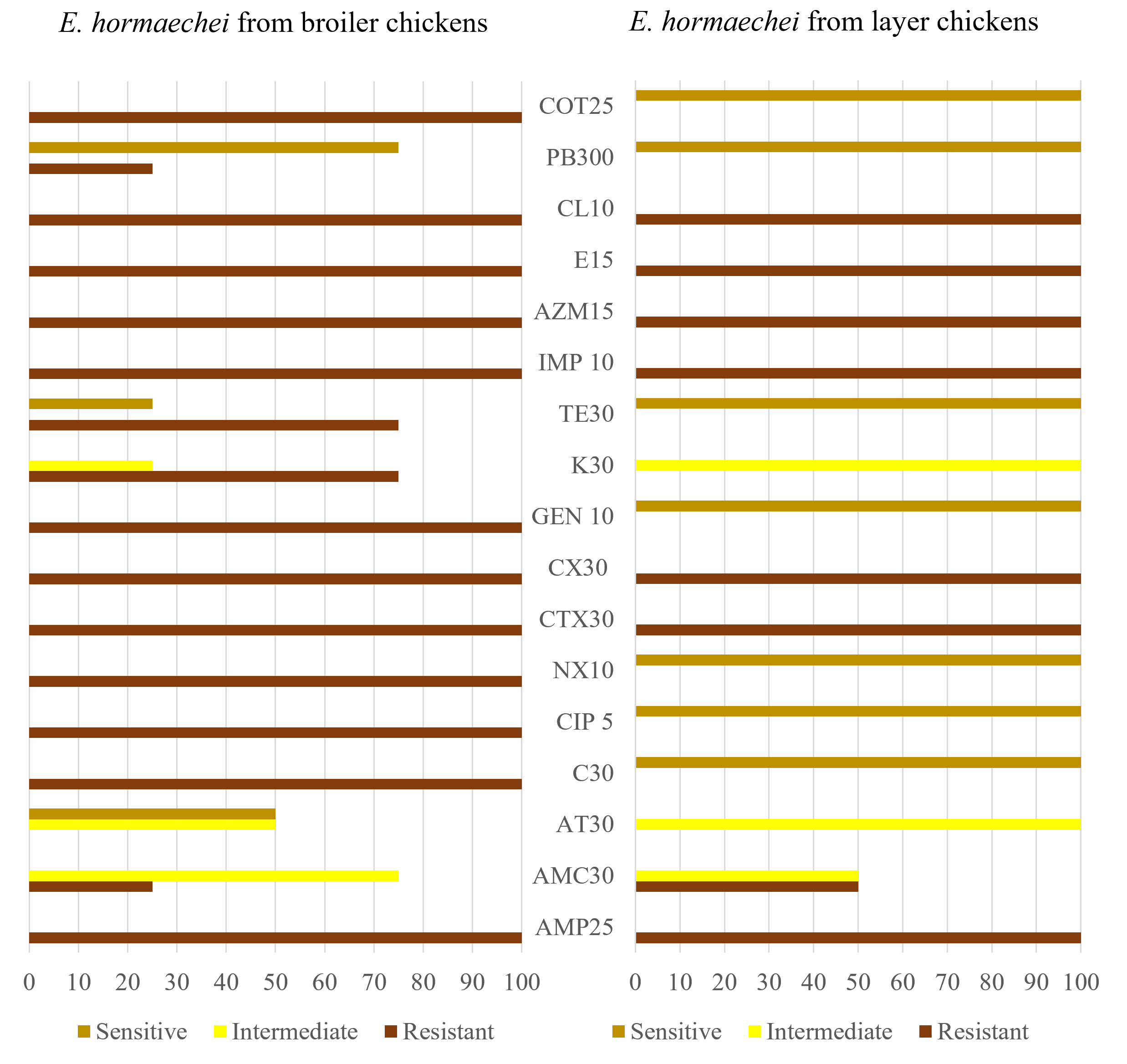

Supplement: S4 Fig — (Ampicillin (AMP25), Amoxicillin-clavulanic acid (AMC 30), Cefotaxime (CTX 30) Cefoxitin (CX 30) Ciprofloxacin (CIP 5) and Norfloxacin (NX 10), Aztreonam (AT 30), Gentamicin (GEN 10), Kanamycin (K 30), Azithromycin (AZM 30) Erythromycin (E 10), Imipenem (IMP 10), Chloramphenicol (C 30), Trimethoprim-Sulfamethoxazole (COT 25), Tetracycline (TE 30), Colistin (CL 10) and Polymyxin B (PB 300)). (TIF) [file pone.0292638.s004.tif]

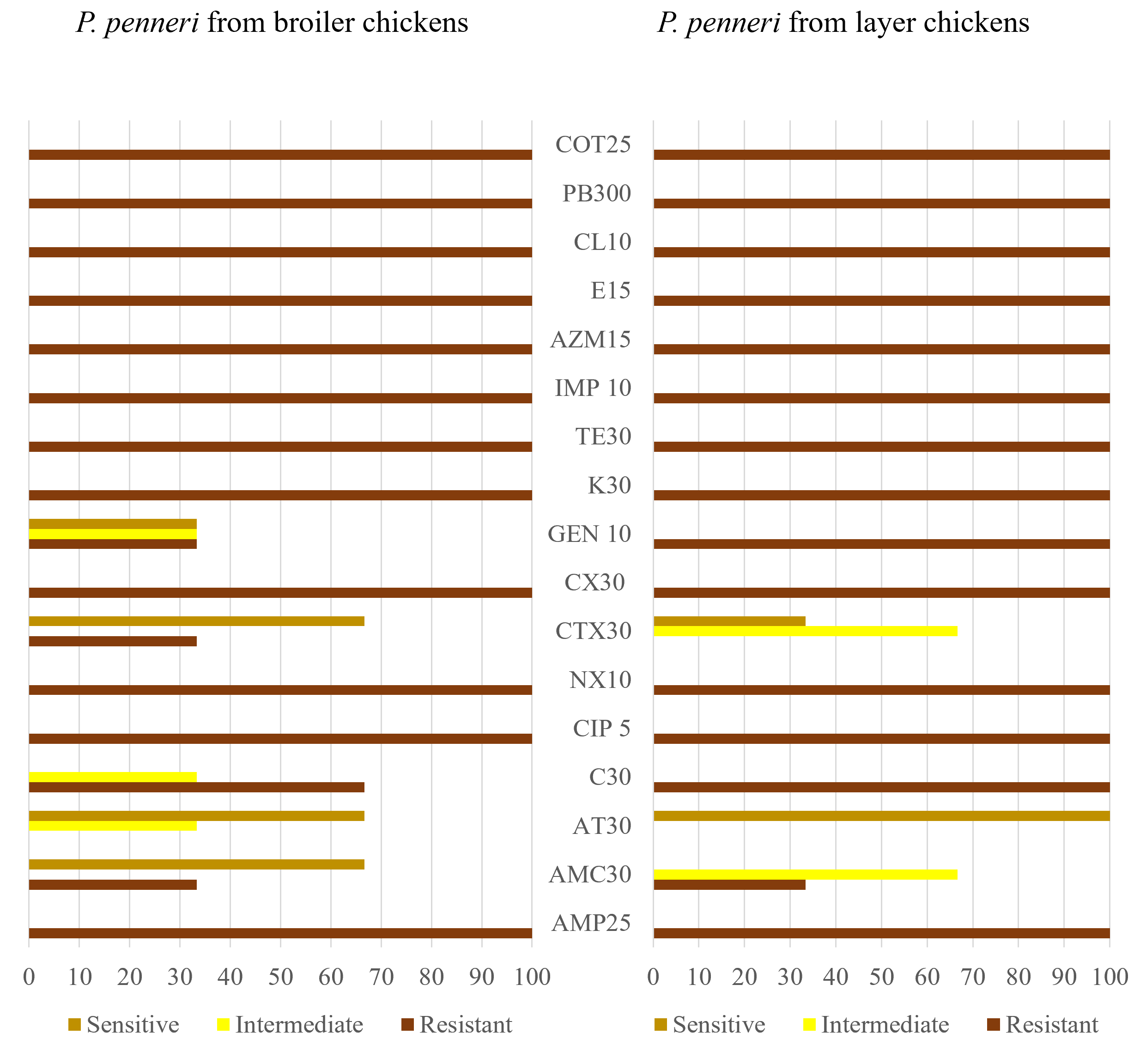

Supplement: S5 Fig — (Ampicillin (AMP25), Amoxicillin-clavulanic acid (AMC 30), Cefotaxime (CTX 30) Cefoxitin (CX 30) Ciprofloxacin (CIP 5) and Norfloxacin (NX 10), Aztreonam (AT 30), Gentamicin (GEN 10), Kanamycin (K 30), Azithromycin (AZM 30) Erythromycin (E 10), Imipenem (IMP 10), Chloramphenicol (C 30), Trimethoprim-Sulfamethoxazole (COT 25), Tetracycline (TE 30), Colistin (CL 10) and Polymyxin B (PB 300)). (TIF) [file pone.0292638.s005.tif]

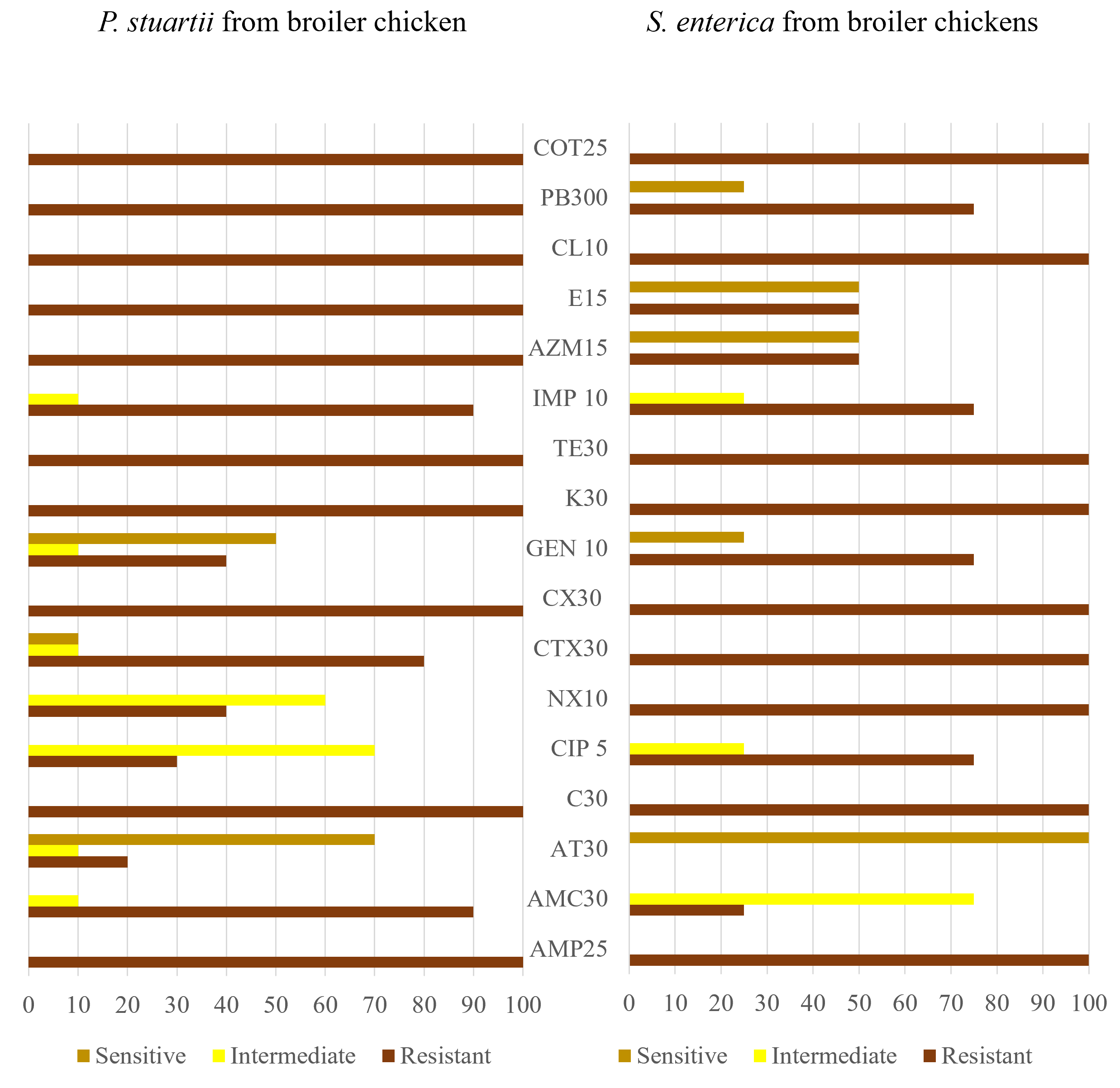

Supplement: S6 Fig — (Ampicillin (AMP25), Amoxicillin-clavulanic acid (AMC 30), Cefotaxime (CTX 30) Cefoxitin (CX 30) Ciprofloxacin (CIP 5) and Norfloxacin (NX 10), Aztreonam (AT 30), Gentamicin (GEN 10), Kanamycin (K 30), Azithromycin (AZM 30) Erythromycin (E 10), Imipenem (IMP 10), Chloramphenicol (C 30), Trimethoprim-Sulfamethoxazole (COT 25), Tetracycline (TE 30), Colistin (CL 10) and Polymyxin B (PB 300)). (TIF) [file pone.0292638.s006.tif]

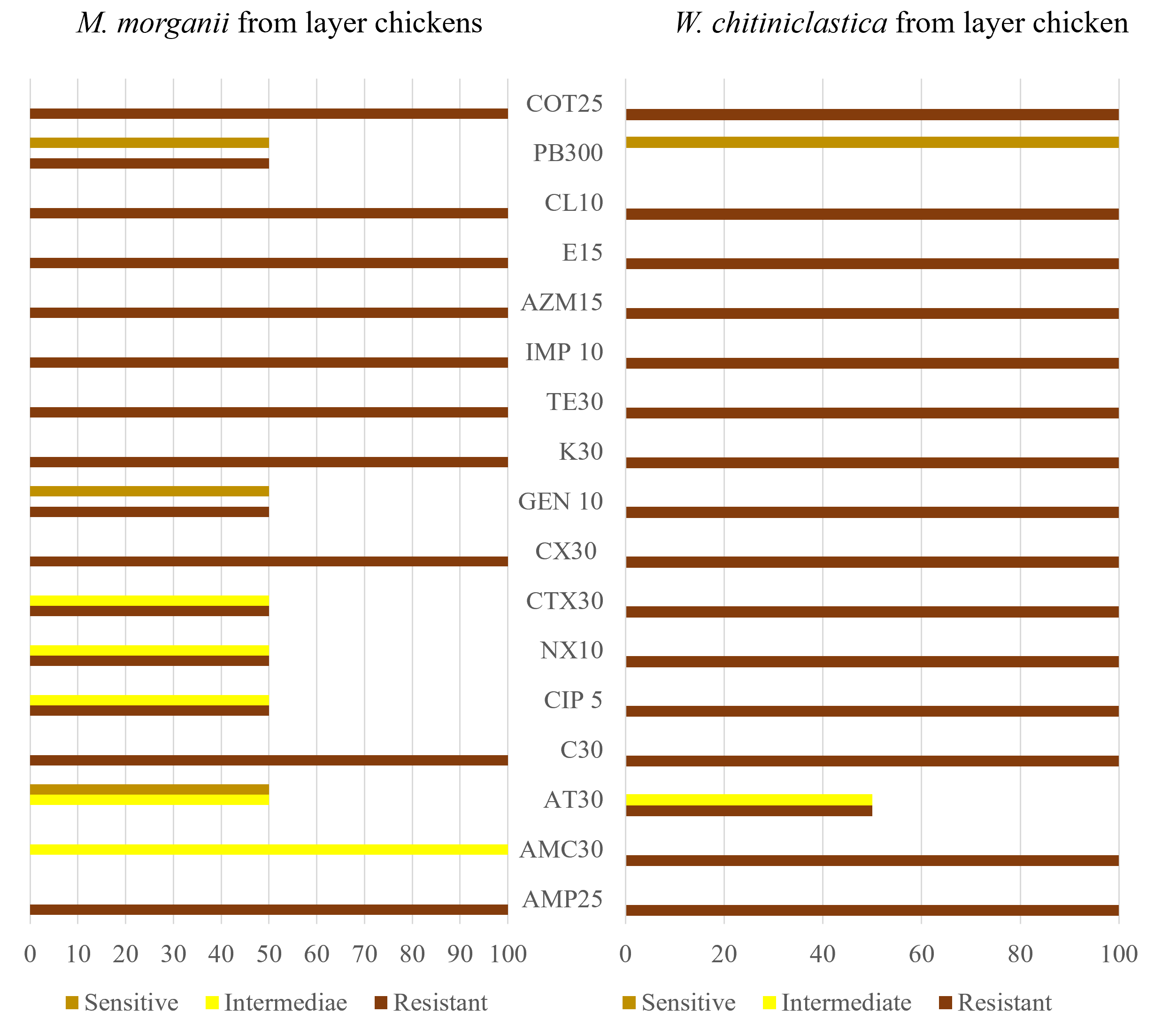

Supplement: S7 Fig — (Ampicillin (AMP25), Amoxicillin-clavulanic acid (AMC 30), Cefotaxime (CTX 30) Cefoxitin (CX 30) Ciprofloxacin (CIP 5) and Norfloxacin (NX 10), Aztreonam (AT 30), Gentamicin (GEN 10), Kanamycin (K 30), Azithromycin (AZM 30) Erythromycin (E 10), Imipenem (IMP 10), Chloramphenicol (C 30), Trimethoprim-Sulfamethoxazole (COT 25), Tetracycline (TE 30), Colistin (CL 10) and Polymyxin B (PB 300)). (TIF) [file pone.0292638.s007.tif]

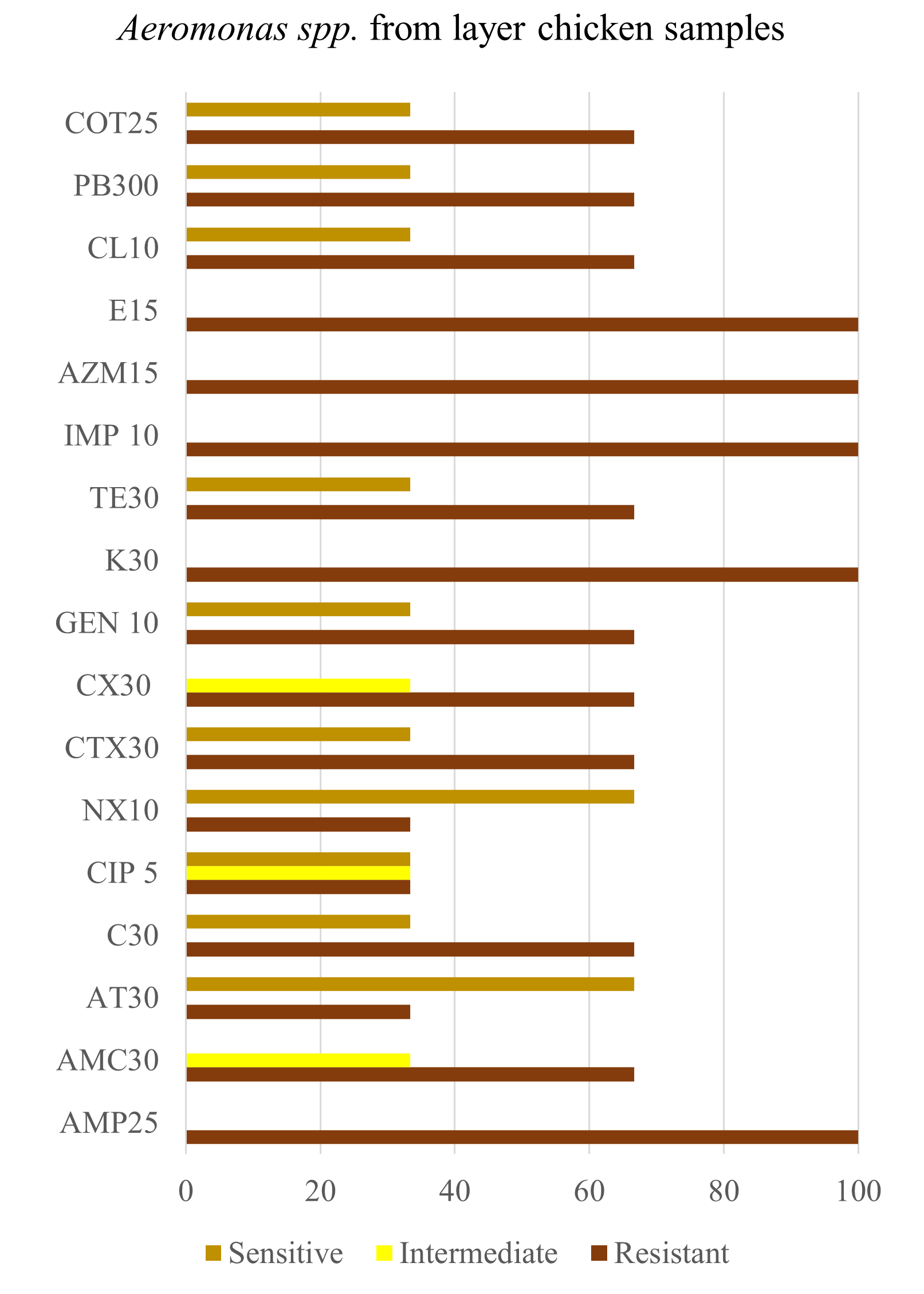

Supplement: S8 Fig — (Ampicillin (AMP25), Amoxicillin-clavulanic acid (AMC 30), Cefotaxime (CTX 30) Cefoxitin (CX 30) Ciprofloxacin (CIP 5) and Norfloxacin (NX 10), Aztreonam (AT 30), Gentamicin (GEN 10), Kanamycin (K 30), Azithromycin (AZM 30) Erythromycin (E 10), Imipenem (IMP 10), Chloramphenicol (C 30), Trimethoprim-Sulfamethoxazole (COT 25), Tetracycline (TE 30), Colistin (CL 10) and Polymyxin B (PB 300)). (TIF) [file pone.0292638.s008.tif]
