# Supplementary material for: Multi-drug resistant (MDR) Gram-negative pathogenic bacteria isolated from poultry in the Noakhali region of Bangladesh
Source: PLoS One. 2024 Aug 1;19(8):e0292638. doi: 10.1371/journal.pone.0292638 (PMC11293736; doi:10.1371/journal.pone.0292638)
Supplement: S1 Table — (DOCX) [file pone.0292638.s009.docx]

**S1 Table: Characteristics of selected farms for sample collection**

|  | **Sample-1 Broiler** | **Sample-2 Layer** | **Sample-3**  **Layer** | **Sample-4 Broiler** | **Sample-5 Broiler** | **Sample-6 Layer** |
| --- | --- | --- | --- | --- | --- | --- |
| **Farm Name** | Shohag Poultry | Nur-hossain agro | Nur-hossain agro | Mousumi Poultry farm | Mousumi Poultry farm | Dipto poultry |
| **Farm location** | Kadirhanif, Sadar, Noakhali. | Puro-char Elahi, Mirzanagar, Sadar, Noakhali. | Puro-char Elahi, Mirzanagar, Sadar, Noakhali. | Kalamia rice mill, Motipur, Sadar, Noakhali. | Kalamia rice mill, Motipur, Sadar, Noakhali. | Bandherhat, Sadar, Noakhali. |
| **Area of the farm** | 6000 Square feet | 10000 square feet | 10000 square feet | 8000 square feet | 8000 square feet | 7000 square feet |
| **Farming capacity** | 1500 | 3000 | 3000 | 2500 | 2500 | 2500 |
| **Nature of the Farm** | Broiler chicken farm | Layer chicken farm | Layer chicken farm | Broiler chicken farm | Broiler chicken farm | Layer chicken farm |
| **Common disease** | Colibacillosis, salmonellosis,  Gumboro,  respiratory disease | Colibacillosis, chronic respiratory disease | Colibacillosis,  chronic respiratory disease | Colibacillosis, salmonellosis and Gumboro | Colibacillosis, salmonellosis and Gumboro | Colibacillosis,  Gumboro. |
