# Supplementary material for: Multi-drug resistant (MDR) Gram-negative pathogenic bacteria isolated from poultry in the Noakhali region of Bangladesh
Source: PLoS One. 2024 Aug 1;19(8):e0292638. doi: 10.1371/journal.pone.0292638 (PMC11293736; doi:10.1371/journal.pone.0292638)
Supplement: S2 Table — (DOCX) [file pone.0292638.s010.docx]

|  | **Sample 1 Broiler** | **Sample 2 Broiler** | **Sample 3**  **Broiler** | **Sample 4 Broiler** | **Sample 5 Broiler** | **Sample 6 Broiler** |
| --- | --- | --- | --- | --- | --- | --- |
| **Age** | 40 days | 280 days | 280 days | 30 days | 30 days | 300 days |
| **Weight** | 1.55 kg | 1.73 kg | 1.9 kg | 1.28 kg | 1.46 kg | 1.65 kg |
| **Food supplied** | Commercial poultry feed | Commercial poultry feed | Commercial poultry feed | Commercial poultry feed | Commercial poultry feed | Commercial poultry feed |
| **Drinking water supplied** | Deep tube-well water | Deep tube-well water | Deep tube-well water | Deep tube-well water | Deep tube-well water | Deep tube-well water |
| **Health condition** | Healthy | Healthy | Healthy | Healthy | Healthy | Healthy |
| **Source of the chicken** | Commercial poultry hatchery | Commercial poultry hatchery | Commercial poultry hatchery | Commercial poultry hatchery | Commercial poultry hatchery | Commercial poultry hatchery |

**S2 Table: Characteristics of poultry chickens**
