# Supplementary material for: Multi-drug resistant (MDR) Gram-negative pathogenic bacteria isolated from poultry in the Noakhali region of Bangladesh
Source: PLoS One. 2024 Aug 1;19(8):e0292638. doi: 10.1371/journal.pone.0292638 (PMC11293736; doi:10.1371/journal.pone.0292638)
Supplement: S3 Table — (DOCX) [file pone.0292638.s011.docx]

**S3 Table: Primers list for different genes used in this study**

| **Gene name** | **Primer name** | **Sequence (5’ to 3’)** | **Primer length** | **Amplicon size (bp)** | **Annealing temp. (**℃) | **Ref.** |
| --- | --- | --- | --- | --- | --- | --- |
| *16S* rRNA | Uni_27F | AGAGTTTGATCCTGGCTCAG | 20 | 1465 | 50 | (1) |
|  | Uni_1492R | GGTTACCTTGTTACGACTT | 19 |  |  |  |
| *rcsA* | *KP*-27F3 | GGATATCTGACCAGTCGG | 18 | 176 | 49 | (2) |
|  | *KP*-27B3 | GGGTTTTGCGTAATGATCTG | 20 |  |  |  |
| *gyr*b | *gyr*b_F | TCCGGCGGTCTGCACGGCGT | 20 | 1100 | 53 | (3) |
|  | *gyr*b_R | TTGTCCGGGTTGTACTCGTC | 20 |  |  |  |
| *bla*CTX-M1 | *blaCTX-M*_F | ATGTGCAGYACCAGTAARGTKATGGC | 26 | 593 | 53 | (4) |
|  | *blaCTX-M*_R | TGGGTRAARTARGTSACCAGAAYCAGCGG | 29 |  |  |  |
| *bla*SHV | *blaSHV*_F | ATGCGTTATATTCGCCTGTG | 20 | 747 | 49 |  |
|  | *blaSHV*_R | TGCTTTGTTATTCGGGCCAA | 20 |  |  |  |
| *bla*TEM | *blaTEM*_F | TCGCCGCATACACTATTCTCAGAATGA | 27 | 445 | 50 |  |
|  | *blaTEM*_R | ACGCTCACCGGCTCCAGATTTAT | 23 |  |  |  |
| *bla*NDM | *blaNDM*-F | GGTTTGGCGATCTGGTTTTC | 20 | 621 | 49 | (5) |
|  | *blaNDM*-R | CGGAATGGCTCATCACGATC | 20 |  |  |  |
| *tetA* | *tet*A-F | GCTACATCCTGCTTGCCTTC | 20 | 201 | 50 | (6) |
|  | *tet*A-R | CATAGATCGCCGTGAAGAGG | 20 |  |  |  |
| *tetB* | *tet*B- F | TTGGTTAGGGGCAAGTTTTG | 20 | 359 | 50 |  |
|  | *tet*B- R | GTAATGGGCCAATAACACCG | 20 |  |  |  |
| *sul1* | *sul*1-F | TTCGGCATTCTGAATCTCAC | 20 | 822 | 49 | (6) |
|  | *sul*1-R | ATGATCTAACCCTCGGTCTC | 20 |  |  |  |
| sul2 | *sul*2-F | CGGCATCGTCAACATAACC | 19 | 625 | 50 |  |
|  | *sul*2-R | GTGTGCGGATGAAGTCAG | 18 |  |  |  |
| *mcr-1* | *CLR-F* | CGGTCAGTCCGTTTGTTC | 18 | 309 | 58 | (7) |
|  | *CLR-R* | CTTGGTCGGTCTGTAGGG | 18 |  |  |  |

**References**

1. Jiang H, Dong H, Zhang G, Yu B, Chapman LR, Fields MW. Microbial diversity in water and sediment of Lake Chaka, an athalassohaline lake in northwestern China. Applied and environmental microbiology. 2006;72(6):3832-45.

2. Dong D, Liu W, Li H, Wang Y, Li X, Zou D, et al. Survey and rapid detection of Klebsiella pneumoniae in clinical samples targeting the rcsA gene in Beijing, China. Frontiers in microbiology. 2015;6:519.

3. Hu M, Wang N, Pan Z, Lu C, Liu Y. Identity and virulence properties of Aeromonas isolates from diseased fish, healthy controls and water environment in China. Letters in Applied Microbiology. 2012;55(3):224-33.

4. Monstein HJ, Östholm‐Balkhed Å, Nilsson M, Nilsson M, Dornbusch K, Nilsson L. Multiplex PCR amplification assay for the detection of blaSHV, blaTEM and blaCTX‐M genes in Enterobacteriaceae. Apmis. 2007;115(12):1400-8.

5. Poirel L, Walsh TR, Cuvillier V, Nordmann P. Multiplex PCR for detection of acquired carbapenemase genes. Diagnostic Microbiology and Infectious Disease. 2011;70(1):119-23. doi: <https://doi.org/10.1016/j.diagmicrobio.2010.12.002>.

6. Fadare FT, Okoh AI. Distribution and molecular characterization of ESBL, pAmpC β-lactamases, and non-β-lactam encoding genes in Enterobacteriaceae isolated from hospital wastewater in Eastern Cape Province, South Africa. Plos one. 2021;16(7):e0254753.

7. Liu Y-Y, Wang Y, Walsh TR, Yi L-X, Zhang R, Spencer J, et al. Emergence of plasmid-mediated colistin resistance mechanism MCR-1 in animals and human beings in China: a microbiological and molecular biological study. The Lancet infectious diseases. 2016;16(2):161-8.
