# Supplementary material for: Multi-drug resistant (MDR) Gram-negative pathogenic bacteria isolated from poultry in the Noakhali region of Bangladesh
Source: PLoS One. 2024 Aug 1;19(8):e0292638. doi: 10.1371/journal.pone.0292638 (PMC11293736; doi:10.1371/journal.pone.0292638)
Supplement: S8 Table — (DOCX) [file pone.0292638.s016.docx]

**S8 Table: PCR mixture preparation for ARG screening via PCR amplification**

| **Reagents** | **Quantity** |
| --- | --- |
| PCR water | 8.5 µl |
| 2X Master mix | 12.5 µl |
| 10 mM Forward primer (27F) | 1 µl |
| 10 mM Reverse primer (1492R) | 1 µl |
| Template DNA | 2 µl |
| Total | 25 µl |
