# Supplementary material for: Multi-drug resistant (MDR) Gram-negative pathogenic bacteria isolated from poultry in the Noakhali region of Bangladesh
Source: PLoS One. 2024 Aug 1;19(8):e0292638. doi: 10.1371/journal.pone.0292638 (PMC11293736; doi:10.1371/journal.pone.0292638)
Supplement: S12 Table — (DOCX) [file pone.0292638.s020.docx]

**S12 Table: PCR condition for *mcr-1 gene* amplification**

| **Steps** | | **Temperature** | **Time** |
| --- | --- | --- | --- |
| 1 cycle | Initial Denaturation | 95 ℃ | 10 min |
| 38 cycles | Denaturation | 95 ℃ | 1 min |
|  | Annealing | 58 ℃ | 1.20 min |
|  | Extension | 72 ℃ | 1.30 min |
| 1 cycle | Final Extension | 72 ℃ | 12 min |
