# Supplementary material for: Multi-drug resistant (MDR) Gram-negative pathogenic bacteria isolated from poultry in the Noakhali region of Bangladesh
Source: PLoS One. 2024 Aug 1;19(8):e0292638. doi: 10.1371/journal.pone.0292638 (PMC11293736; doi:10.1371/journal.pone.0292638)
Supplement: S13 Table — (DOCX) [file pone.0292638.s021.docx]

**S13 Table: Standard Zone of Inhibition (ZOI) data to interpret antibiogram result of bacteria according to CLSI 2018 (8)**

|  | Standard values in mm for *Enterobacteriaceae* | | | Standard values in mm for *Aeromonas spp.* | | |
| --- | --- | --- | --- | --- | --- | --- |
| Antibiotics | Resistant | Intermediate | Susceptible | Resistant | Intermediate | Susceptible |
| Ampicillin (AMP25) | ≤13 | 14-16 | ≥17 |  |  |  |
| Amoxycillin clavulanic acid (AMC30) | ≤13 | 14-17 | ≥18 |  |  |  |
| Aztreonam (AT 30) | ≤17 | 18-20 | ≥21 | ≤17 | 18-20 | ≥21 |
| Cefotaxime (CTX 30) | ≤22 | 23-25 | ≥26 | ≤22 | 23-25 | ≥26 |
| Cefoxitin (CX 30) | ≤14 | 15-17 | ≥18 | ≤14 | 15-17 | ≥18 |
| Norfloxacin (NX 10) | ≤12 | 13-16 | ≥17 |  |  |  |
| Ciprofloxacin (CIP 5) | ≤20 | 21-30 | ≥31 | ≤15 | 16-20 | ≥15 |
| Chloramphenicol (C30) | ≤12 | 13-17 | ≥18 | ≤12 | 13-17 | ≥18 |
| Imipenem (IMP 10) | ≤19 | 20-22 | ≥23 | ≤19 | 20-22 | ≥23 |
| Tetracycline (TE 30) | ≤18 | 19-24 | ≥25 | ≤11 | 12-14 | ≥15 |
| Co- Trimoxazole (COT 25) | ≤10 | 11-15 | ≥16 | ≤10 | 11-15 | ≥16 |
| Azithromycine (AZM30) | ≤13 | 14-17 | ≥18 |  |  |  |
| Erythromycin (E15) |  |  |  |  |  |  |
| Gentamicin (GEN 10) | ≤12 | 13-14 | ≥15 | ≤12 | 13-14 | ≥15 |
| Kanamycin (K 30) | ≤13 | 14-17 | ≥18 |  |  |  |

**References**

8. Wayne P. CLSI Performance Standards for Antimicrobial Susceptibility Testing. CLSI supplements M. 2020;100.
