# Supplementary material for: Multi-drug resistant (MDR) Gram-negative pathogenic bacteria isolated from poultry in the Noakhali region of Bangladesh
Source: PLoS One. 2024 Aug 1;19(8):e0292638. doi: 10.1371/journal.pone.0292638 (PMC11293736; doi:10.1371/journal.pone.0292638)
Supplement: S1 File — (DOCX) [file pone.0292638.s022.docx]

**S1** **Sanger di-deoxy sequencing data**

1. **Sample_12: *P. stuartii***


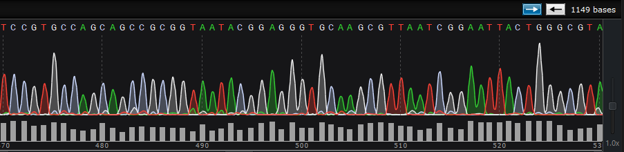


**Sanger di-deoxy sequencing chromatogram image of Sample_12 (*P. stuartii*)**

**Full sequence obtained from sanger sequencing**

AKCGGCAGCTACACATGCAAGTCGTAACAAGGTAACCYAAACTTGYTTCYCSYTGACKAGCGGCGGAGGGRTGGGTAATGTMYGGGGATCTGCCCGATAKAGGGGGATAACTACTGKAMACGGKGGCTAATACCGYATMCTCTCTTAGGAGCAAAGCAGGGGACCTTCGGGCCTTGCGCTGTCGGATGAACCCATATGGGATTAGCTAGTAGGTAAGGTAATGGCTTACCTAGGCGACGAWCCCTAGCTGGTCTGAGAGGATGATCAGCCACACTGGGACTGAGACACGGCCCAGACTCCTACGGGAGGCAGCAGTGGGGAATATTGCACAATGGGCGCAAGCCTGATGCAGCCATGCCGCGTGTATGAAGAAGGCCCTAGGGTTGTAAAGTACTTTCAGTCGGGAGGAAGGCGTTGATGTTAATACCATCARCGATTGACGTTACCGACAGAAGAAGCACCGGCTAACTCCGTGCCAGCAGCCGCGGTAATACGGAGGGTGCAAGCGTTAATCGGAATTACTGGGCGTAAAGCGCACGCAGGCGGTTAATTAAGTTAGATGTGAAATCCCCGGGCTTAACCTGGGAATGGCATCTAAGACTGGTTAGCTAGAGTCTTGTAGAGGGGGGTAGAATTCCATGTGTAGCGGTGAAATGCGTAGAGATGTGGAGGAATACCGGTGGCGAAGGCGGCCCCCTGGACAAAGACTGACGCTCAGGTGCGAAAGCGTGGGGAGCAAACAGGATTAGATACCCTGGTAGTCCACGCTGTAAACGATGTCGATTTGGAGGTTGTTCCCTAGAGGAGTGGCTTCCGGAGCTAACGCGTTAAATCGACCGCCTGGGGAGTACGGCCGCAAGGTTAAAACTCAAATGAATTGACGGGGGCCCGCACAAGCGGTGGAGCATGTGGTTTAATTCGATGCAACGCGAAGAACCTTACCTACTCTTGACATCCAGAGAATTTGRGCAGAGATGCTKWAGTGCCTTCGGGAACTCTGAGACAGGTGCTGCATGGCTGTCGTCAGCTCGTGTTGTGAAATGTTGGTTAAGTCCCGCAACGAGCGCAACCCTTATCCCTTTGTTGCCAGCGATTCGGTYCGGCAACTCAAGGAGACTGCCGGTGCATAAACCGGAAAGGAAGGTKGGG

**Selected sequence for blast run (144-979 bp)**

CTTAGGAGCAAAGCAGGGGACCTTCGGGCCTTGCGCTGTCGGATGAACCCATATGGGATTAGCTAGTAGGTAAGGTAATGGCTTACCTAGGCGACGAWCCCTAGCTGGTCTGAGAGGATGATCAGCCACACTGGGACTGAGACACGGCCCAGACTCCTACGGGAGGCAGCAGTGGGGAATATTGCACAATGGGCGCAAGCCTGATGCAGCCATGCCGCGTGTATGAAGAAGGCCCTAGGGTTGTAAAGTACTTTCAGTCGGGAGGAAGGCGTTGATGTTAATACCATCARCGATTGACGTTACCGACAGAAGAAGCACCGGCTAACTCCGTGCCAGCAGCCGCGGTAATACGGAGGGTGCAAGCGTTAATCGGAATTACTGGGCGTAAAGCGCACGCAGGCGGTTAATTAAGTTAGATGTGAAATCCCCGGGCTTAACCTGGGAATGGCATCTAAGACTGGTTAGCTAGAGTCTTGTAGAGGGGGGTAGAATTCCATGTGTAGCGGTGAAATGCGTAGAGATGTGGAGGAATACCGGTGGCGAAGGCGGCCCCCTGGACAAAGACTGACGCTCAGGTGCGAAAGCGTGGGGAGCAAACAGGATTAGATACCCTGGTAGTCCACGCTGTAAACGATGTCGATTTGGAGGTTGTTCCCTAGAGGAGTGGCTTCCGGAGCTAACGCGTTAAATCGACCGCCTGGGGAGTACGGCCGCAAGGTTAAAACTCAAATGAATTGACGGGGGCCCGCACAAGCGGTGGAGCATGTGGTTTAATTCGATGCAACGCGAAGAACCTTACCTACTCTTGACATCCAGAGAATTTGRGCAGAGATGCT

**Blast result of *P. Stuartii***

*Providencia stuartii* strain MF1 16S ribosomal RNA gene, partial sequence

Sequence ID: MT023702.1 Length: 1545

Range 1: 187 to 1021

Score:1530 bits (828), Expect:0.0, Identities:833/836(99%), Gaps:1/836 (0%), Strand: Plus/Plus

**Query 1 CTTAGGAGCAAAGCAGGGGACCTTCGGGCCTTGCGCTGTCGGATGAACCCATATGGGATT 60**

**||||||||||||||||||||||||||||||||||||||||||||||||||||||||||||**

**Sbjct 187 CTTAGGAGCAAAGCAGGGGACCTTCGGGCCTTGCGCTGTCGGATGAACCCATATGGGATT 246**

**Query 61 AGCTAGTAGGTAAGGTAATGGCTTACCTAGGCGACGAWCCCTAGCTGGTCTGAGAGGATG 120**

**||||||||||||||||||||||||||||||||||||| ||||||||||||||||||||||**

**Sbjct 247 AGCTAGTAGGTAAGGTAATGGCTTACCTAGGCGACGATCCCTAGCTGGTCTGAGAGGATG 306**

**Query 121 ATCAGCCACACTGGGACTGAGACACGGCCCAGACTCCTACGGGAGGCAGCAGTGGGGAAT 180**

**||||||||||||||||||||||||||||||||||||||||||||||||||||||||||||**

**Sbjct 307 ATCAGCCACACTGGGACTGAGACACGGCCCAGACTCCTACGGGAGGCAGCAGTGGGGAAT 366**

**Query 181 ATTGCACAATGGGCGCAAGCCTGATGCAGCCATGCCGCGTGTATGAAGAAGGCCCTAGGG 240**

**||||||||||||||||||||||||||||||||||||||||||||||||||||||||||||**

**Sbjct 367 ATTGCACAATGGGCGCAAGCCTGATGCAGCCATGCCGCGTGTATGAAGAAGGCCCTAGGG 426**

**Query 241 TTGTAAAGTACTTTCAGTCGGGAGGAAGGCGTTGATGTTAATACCATCARCGATTGACGT 300**

**||||||||||||||||||||||||||||||||||||||||||||||||| ||||||||||**

**Sbjct 427 TTGTAAAGTACTTTCAGTCGGGAGGAAGGCGTTGATGTTAATACCATCAACGATTGACGT 486**

**Query 301 TACCGACAGAAGAAGCACCGGCTAACTCCGTGCCAGCAGCCGCGGTAATACGGAGGGTGC 360**

**||||||||||||||||||||||||||||||||||||||||||||||||||||||||||||**

**Sbjct 487 TACCGACAGAAGAAGCACCGGCTAACTCCGTGCCAGCAGCCGCGGTAATACGGAGGGTGC 546**

**Query 361 AAGCGTTAATCGGAATTACTGGGCGTAAAGCGCACGCAGGCGGTTAATTAAGTTAGATGT 420**

**||||||||||||||||||||||||||||||||||||||||||||||||||||||||||||**

**Sbjct 547 AAGCGTTAATCGGAATTACTGGGCGTAAAGCGCACGCAGGCGGTTAATTAAGTTAGATGT 606**

**Query 421 GAAATCCCCGGGCTTAACCTGGGAATGGCATCTAAGACTGGTTAGCTAGAGTCTTGTAGA 480**

**||||||||||||||||||||||||||||||||||||||||||||||||||||||||||||**

**Sbjct 607 GAAATCCCCGGGCTTAACCTGGGAATGGCATCTAAGACTGGTTAGCTAGAGTCTTGTAGA 666**

**Query 481 GGGGGGTAGAATTCCATGTGTAGCGGTGAAATGCGTAGAGATGTGGAGGAATACCGGTGG 540**

**||||||||||||||||||||||||||||||||||||||||||||||||||||||||||||**

**Sbjct 667 GGGGGGTAGAATTCCATGTGTAGCGGTGAAATGCGTAGAGATGTGGAGGAATACCGGTGG 726**

**Query 541 CGAAGGCGGCCCCCTGGACAAAGACTGACGCTCAGGTGCGAAAGCGTGGGGAGCAAACAG 600**

**||||||||||||||||||||||||||||||||||||||||||||||||||||||||||||**

**Sbjct 727 CGAAGGCGGCCCCCTGGACAAAGACTGACGCTCAGGTGCGAAAGCGTGGGGAGCAAACAG 786**

**Query 601 GATTAGATACCCTGGTAGTCCACGCTGTAAACGATGTCGATTTGGAGGTTGTTCCCTAGA 660**

**||||||||||||||||||||||||||||||||||||||||||||||||||||||||||||**

**Sbjct 787 GATTAGATACCCTGGTAGTCCACGCTGTAAACGATGTCGATTTGGAGGTTGTTCCCTAGA 846**

**Query 661 GGAGTGGCTTCCGGAGCTAACGCGTTAAATCGACCGCCTGGGGAGTACGGCCGCAAGGTT 720**

**||||||||||||||||||||||||||||||||||||||||||||||||||||||||||||**

**Sbjct 847 GGAGTGGCTTCCGGAGCTAACGCGTTAAATCGACCGCCTGGGGAGTACGGCCGCAAGGTT 906**

**Query 721 AAAACTCAAATGAATTGACGGGGGCCCGCACAAGCGGTGGAGCATGTGGTTTAATTCGAT 780**

**||||||||||||||||||||||||||||||||||||||||||||||||||||||||||||**

**Sbjct 907 AAAACTCAAATGAATTGACGGGGGCCCGCACAAGCGGTGGAGCATGTGGTTTAATTCGAT 966**

**Query 781 GCAACGCGAAGAACCTTACCTACTCTTGACATCCAGAGAATTTGRGCAGAGATGCT 836**

**|||||||||||||||||||||||||||||||||||||||||||| |||||||||||**

**Sbjct 967 GCAACGCGAAGAACCTTACCTACTCTTGACATCCAGAGAATTTG-GCAGAGATGCT 1021**

1. **Sample_13: *E. hormaechei***


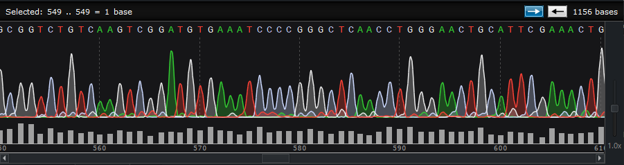


**Sanger di-deoxy sequencing chromatogram image of Sample_13 (*E. hormaechei*)**

**Full sequence obtained from sanger sequencing**

AGMCGCRRGCGTAACACATGCAAGTCGAACGGTRACAGCGCAAGCAGCTTGCTGCTTCGCTGACSAGTGGCGGACGGGTGAGTARTGTCTGGGAAACTGCCTGATGGAGGGGGATAACTACTGGAAACGGTAGCTAATACCGCATAACGTCGCRAGACCAAAGAGGGGGACCTTCGGGCCTCTTGCCATCGGATGTGCCCAGATGGGATTAGCTAGTAGGTGGGGTAACGGCTCACCTAGGCGACGATCCCTAGCTGGTCTGAGAGGATGACCRGCCACRCTGGAACTGAGACACGGTCCAGACTCCTACGGGAGGCAGCAGTGGGGAATATTGCACAATGGGCGCAAGCCTGATGCAGCCATGCCGCGKGTATGAAGAAGGCCTTCGGGTTGTAAAGTACTTTCAGCGGGGAGGAAGGCGATRAGGTTAATAACCTTGTCGATTGACGTTACCCGCAGAAGAAGCACCGGCTAACTCCGTGCCAGCAGCCGCGGTAATACGGAGGGTGCAAGCGTTAATCGGAATTACTGGGCGTAAAGCGCACGCAGGCGGTCTGTCAAGTCGGATGTGAAATCCCCGGGCTCAACCTGGGAACTGCATTCGAAACTGGCAGGCTAGAGTCTTGTAGAGGGGGGTAGAATTCCAGGTGTAGCGGTGAAATGCGTAGAGATCTGGAGGAATACCGGTGGCGAAGGCGGCCCCCTGGACAAAGACTGACGCTCAGGTGCGAAAGCGTGGGGAGCAAACAGGATTAGATACCCTGGTAGTCCACGCCGTAAACGATGTCGACTTGGAGGTTGTGCCCTTGAGGCGTGGCTTCCGGAGCTAACGCGTTAAGTCGACCGCCTGGGGAGTACGGCCGCAAGGTTAAAACTCAAATGAATTGACGGGGGCCCGCACAAGCGGTGGAGCATGTGGTTTAATTCGATGCAACGCGAAGAACCTTACCTACTCTTGACATCCAGAGAACTTAGCAGAGATGCWTTGGTGCCTTCGGGAACTCTGAGACAGGTGCTGCATGGCTGTCGTCAGCTCGTGTTGTGAAATGTTGGGTTAAGTCCGCAACGAGCGCAACCCTTATCCTTTGGTGGCCAGCGGGTTCAGTCGGTACTCAAGGAGACTGCCAGCTGATAACCTGGGAAGGAATGGTKGGGG

**Selected sequence for blast run (87-1053 bp)**

GTCTGGGAAACTGCCTGATGGAGGGGGATAACTACTGGAAACGGTAGCTAATACCGCATAACGTCGCRAGACCAAAGAGGGGGACCTTCGGGCCTCTTGCCATCGGATGTGCCCAGATGGGATTAGCTAGTAGGTGGGGTAACGGCTCACCTAGGCGACGATCCCTAGCTGGTCTGAGAGGATGACCRGCCACRCTGGAACTGAGACACGGTCCAGACTCCTACGGGAGGCAGCAGTGGGGAATATTGCACAATGGGCGCAAGCCTGATGCAGCCATGCCGCGKGTATGAAGAAGGCCTTCGGGTTGTAAAGTACTTTCAGCGGGGAGGAAGGCGATRAGGTTAATAACCTTGTCGATTGACGTTACCCGCAGAAGAAGCACCGGCTAACTCCGTGCCAGCAGCCGCGGTAATACGGAGGGTGCAAGCGTTAATCGGAATTACTGGGCGTAAAGCGCACGCAGGCGGTCTGTCAAGTCGGATGTGAAATCCCCGGGCTCAACCTGGGAACTGCATTCGAAACTGGCAGGCTAGAGTCTTGTAGAGGGGGGTAGAATTCCAGGTGTAGCGGTGAAATGCGTAGAGATCTGGAGGAATACCGGTGGCGAAGGCGGCCCCCTGGACAAAGACTGACGCTCAGGTGCGAAAGCGTGGGGAGCAAACAGGATTAGATACCCTGGTAGTCCACGCCGTAAACGATGTCGACTTGGAGGTTGTGCCCTTGAGGCGTGGCTTCCGGAGCTAACGCGTTAAGTCGACCGCCTGGGGAGTACGGCCGCAAGGTTAAAACTCAAATGAATTGACGGGGGCCCGCACAAGCGGTGGAGCATGTGGTTTAATTCGATGCAACGCGAAGAACCTTACCTACTCTTGACATCCAGAGAACTTAGCAGAGATGCWTTGGTGCCTTCGGGAACTCTGAGACAGGTGCTGCATGGCTGTCGTCAGCTCGTGTTGTGAAATGTTGG

**Blast result of *E. hormaechei***

*Enterobacter hormaechei* strain AR9N 16S ribosomal RNA gene, partial sequence

Sequence ID: MT509859.1 Length: 1305

Range 1: 76 to 1042

Score:1764 bits (955), Expect: 0.0, Identities:961/967(99%), Gaps:0/967 (0%), Strand: Plus/Plus

Query 1 GTCTGGGAAACTGCCTGATGGAGGGGGATAACTACTGGAAACGGTAGCTAATACCGCATA 60

||||||||||||||||||||||||||||||||||||||||||||||||||||||||||||

Sbjct 76 GTCTGGGAAACTGCCTGATGGAGGGGGATAACTACTGGAAACGGTAGCTAATACCGCATA 135

Query 61 ACGTCGCRAGACCAAAGAGGGGGACCTTCGGGCCTCTTGCCATCGGATGTGCCCAGATGG 120

||||||| ||||||||||||||||||||||||||||||||||||||||||||||||||||

Sbjct 136 ACGTCGCAAGACCAAAGAGGGGGACCTTCGGGCCTCTTGCCATCGGATGTGCCCAGATGG 195

Query 121 GATTAGCTAGTAGGTGGGGTAACGGCTCACCTAGGCGACGATCCCTAGCTGGTCTGAGAG 180

||||||||||||||||||||||||||||||||||||||||||||||||||||||||||||

Sbjct 196 GATTAGCTAGTAGGTGGGGTAACGGCTCACCTAGGCGACGATCCCTAGCTGGTCTGAGAG 255

Query 181 GATGACCRGCCACRCTGGAACTGAGACACGGTCCAGACTCCTACGGGAGGCAGCAGTGGG 240

||||||| ||||| ||||||||||||||||||||||||||||||||||||||||||||||

Sbjct 256 GATGACCAGCCACACTGGAACTGAGACACGGTCCAGACTCCTACGGGAGGCAGCAGTGGG 315

Query 241 GAATATTGCACAATGGGCGCAAGCCTGATGCAGCCATGCCGCGKGTATGAAGAAGGCCTT 300

||||||||||||||||||||||||||||||||||||||||||| ||||||||||||||||

Sbjct 316 GAATATTGCACAATGGGCGCAAGCCTGATGCAGCCATGCCGCGTGTATGAAGAAGGCCTT 375

Query 301 CGGGTTGTAAAGTACTTTCAGCGGGGAGGAAGGCGATRAGGTTAATAACCTTGTCGATTG 360

||||||||||||||||||||||||||||||||||||| ||||||||||||||||||||||

Sbjct 376 CGGGTTGTAAAGTACTTTCAGCGGGGAGGAAGGCGATAAGGTTAATAACCTTGTCGATTG 435

Query 361 ACGTTACCCGCAGAAGAAGCACCGGCTAACTCCGTGCCAGCAGCCGCGGTAATACGGAGG 420

||||||||||||||||||||||||||||||||||||||||||||||||||||||||||||

Sbjct 436 ACGTTACCCGCAGAAGAAGCACCGGCTAACTCCGTGCCAGCAGCCGCGGTAATACGGAGG 495

Query 421 GTGCAAGCGTTAATCGGAATTACTGGGCGTAAAGCGCACGCAGGCGGTCTGTCAAGTCGG 480

||||||||||||||||||||||||||||||||||||||||||||||||||||||||||||

Sbjct 496 GTGCAAGCGTTAATCGGAATTACTGGGCGTAAAGCGCACGCAGGCGGTCTGTCAAGTCGG 555

Query 481 ATGTGAAATCCCCGGGCTCAACCTGGGAACTGCATTCGAAACTGGCAGGCTAGAGTCTTG 540

||||||||||||||||||||||||||||||||||||||||||||||||||||||||||||

Sbjct 556 ATGTGAAATCCCCGGGCTCAACCTGGGAACTGCATTCGAAACTGGCAGGCTAGAGTCTTG 615

Query 541 TAGAGGGGGGTAGAATTCCAGGTGTAGCGGTGAAATGCGTAGAGATCTGGAGGAATACCG 600

||||||||||||||||||||||||||||||||||||||||||||||||||||||||||||

Sbjct 616 TAGAGGGGGGTAGAATTCCAGGTGTAGCGGTGAAATGCGTAGAGATCTGGAGGAATACCG 675

Query 601 GTGGCGAAGGCGGCCCCCTGGACAAAGACTGACGCTCAGGTGCGAAAGCGTGGGGAGCAA 660

||||||||||||||||||||||||||||||||||||||||||||||||||||||||||||

Sbjct 676 GTGGCGAAGGCGGCCCCCTGGACAAAGACTGACGCTCAGGTGCGAAAGCGTGGGGAGCAA 735

Query 661 ACAGGATTAGATACCCTGGTAGTCCACGCCGTAAACGATGTCGACTTGGAGGTTGTGCCC 720

||||||||||||||||||||||||||||||||||||||||||||||||||||||||||||

Sbjct 736 ACAGGATTAGATACCCTGGTAGTCCACGCCGTAAACGATGTCGACTTGGAGGTTGTGCCC 795

Query 721 TTGAGGCGTGGCTTCCGGAGCTAACGCGTTAAGTCGACCGCCTGGGGAGTACGGCCGCAA 780

||||||||||||||||||||||||||||||||||||||||||||||||||||||||||||

Sbjct 796 TTGAGGCGTGGCTTCCGGAGCTAACGCGTTAAGTCGACCGCCTGGGGAGTACGGCCGCAA 855

Query 781 GGTTAAAACTCAAATGAATTGACGGGGGCCCGCACAAGCGGTGGAGCATGTGGTTTAATT 840

||||||||||||||||||||||||||||||||||||||||||||||||||||||||||||

Sbjct 856 GGTTAAAACTCAAATGAATTGACGGGGGCCCGCACAAGCGGTGGAGCATGTGGTTTAATT 915

Query 841 CGATGCAACGCGAAGAACCTTACCTACTCTTGACATCCAGAGAACTTAGCAGAGATGCWT 900

|||||||||||||||||||||||||||||||||||||||||||||||||||||||||| |

Sbjct 916 CGATGCAACGCGAAGAACCTTACCTACTCTTGACATCCAGAGAACTTAGCAGAGATGCTT 975

Query 901 TGGTGCCTTCGGGAACTCTGAGACAGGTGCTGCATGGCTGTCGTCAGCTCGTGTTGTGAA 960

||||||||||||||||||||||||||||||||||||||||||||||||||||||||||||

Sbjct 976 TGGTGCCTTCGGGAACTCTGAGACAGGTGCTGCATGGCTGTCGTCAGCTCGTGTTGTGAA 1035

Query 961 ATGTTGG 967

|||||||

Sbjct 1036 ATGTTGG 1042

1. **Sample_15: *W. chitiniclastica***


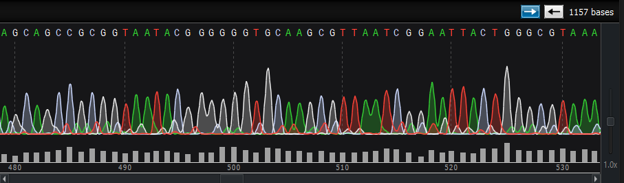


**Sanger di-deoxy sequencing chromatogram image of Sample_15: (*W. chitiniclastica*)**

**Full sequence obtained from sanger sequencing**

CKGCGGGTAACTCCATGCAGTCGTACARGGYAACCGAAAKTKTACTTCTTCGGCGTCGAGTGGCGGACGGGTGAGKAATGCATAGGAATCTGCCCTATAGTTTGGGATAACTACTCGAAAGAGTGGCTAATACCGGATGTGGACTACSGTTTARAGCARGGGACCTTCSGGCCTTGCKCTATAGGATGAGCCTATGTGGGATTAGCTAGTTGGTGAGGTAAAGGCTCACCAARGCRACSATCTCTAGCTGGTCTGARAGGATGATCASCCACACTGGGACTGAGACACGGCCCASACTCCTACGGGAGGCAGCAGTGGGGAATATTGGACAATGGGCGGAAGCCTGATCCAGCAATACCGCGTGTGTGAAGAAGGCCTTCKGGTTGTAAAGCACTTTTGTCAGGRAGAATGGCAGTTGAGGTTAATACCCTTAKCTGTTGATGTTACCTGAAGAATAAGCACCGGCTAACTCCGTGCCAGCAGCCGCGGTAATACGGGGGGTGCAAGCGTTAATCGGAATTACTGGGCGTAAAGGGCGCGTASGCGGTTACTTAAGTTAGATGTGAAAGCCCCGGGCTTAACCTGGGAATTGCATTTAATACTGGGTAACTAGAGTGTGGTAGAGAGTAGCGGAATTTCTGGTGTARCGGTGAAATGCRTAKATATCAGAASGAACACCAATGGCGAASGCAGCTGCTTGGRCCRACACTGACGCTGARGCGCGAAAGCGTGGGGAGCAAACAGGATTASATACCCTGGTAGTCCACGCTGTAAACGATGACAACTTATTGATGGGAGGGTTACCTTTCGTTGATGTAGCTAACGTGTTAAGTTGTCCGCCTGGGGAGTACGGTCGCAAGGCTGAAACTCAAAGGAATTGACGGGGACCCGCACAAGCGGTGGAGCATGTGGTTTAATTCGATGCAACGMGMAKAACCTTACCTGGTCTTGACATCTACAKAACTWTCCAGAGATGGATTGTGCCTWCWGGAACTGTAAGACAGGTGCTGCATGGCTGTCGTCAGCTCGTGWCRTGAGATGTAGGGTTAAGTCCGCAACGAAGCGCTATCCTGTCCGTTATTTGCCAGCACATGAATGACTGGGACTTCGGGCAGACTGACCGAATGATAAGTCGATGAGTRGGCATGAYCGTTTCA

**Selected sequence for blast run (270-1082 bp)**

CACACTGGGACTGAGACACGGCCCASACTCCTACGGGAGGCAGCAGTGGGGAATATTGGACAATGGGCGGAAGCCTGATCCAGCAATACCGCGTGTGTGAAGAAGGCCTTCKGGTTGTAAAGCACTTTTGTCAGGRAGAATGGCAGTTGAGGTTAATACCCTTAKCTGTTGATGTTACCTGAAGAATAAGCACCGGCTAACTCCGTGCCAGCAGCCGCGGTAATACGGGGGGTGCAAGCGTTAATCGGAATTACTGGGCGTAAAGGGCGCGTASGCGGTTACTTAAGTTAGATGTGAAAGCCCCGGGCTTAACCTGGGAATTGCATTTAATACTGGGTAACTAGAGTGTGGTAGAGAGTAGCGGAATTTCTGGTGTARCGGTGAAATGCRTAKATATCAGAASGAACACCAATGGCGAASGCAGCTGCTTGGRCCRACACTGACGCTGARGCGCGAAAGCGTGGGGAGCAAACAGGATTASATACCCTGGTAGTCCACGCTGTAAACGATGACAACTTATTGATGGGAGGGTTACCTTTCGTTGATGTAGCTAACGTGTTAAGTTGTCCGCCTGGGGAGTACGGTCGCAAGGCTGAAACTCAAAGGAATTGACGGGGACCCGCACAAGCGGTGGAGCATGTGGTTTAATTCGATGCAACGMGMAKAACCTTACCTGGTCTTGACATCTACAKAACTWTCCAGAGATGGATTGTGCCTWCWGGAACTGTAAGACAGGTGCTGCATGGCTGTCGTCAGCTCGTGWCRTGAGATGTAGGGTTAAGTCCGCAACGAAGCGCTATCCTGTCCGTTATT

**Blast result of *W. chitiniclastica***

*Wohlfahrtiimonas chitiniclastica* strain NY02 16S ribosomal RNA gene, partial sequence

Sequence ID: MF037998.1 Length: 1482

Range 1: 301 to 1114

Score: 1371 bits (742), Expect: 0.0, Identities: 785/816 (96%), Gaps: 5/816 (0%), Strand: Plus/Plus

Query 1 CACACTGGGACTGAGACACGGCCCASACTCCTACGGGAGGCAGCAGTGGGGAATATTGGA 60

||||||||||||||||||||||||| ||||||||||||||||||||||||||||||||||

Sbjct 301 CACACTGGGACTGAGACACGGCCCAGACTCCTACGGGAGGCAGCAGTGGGGAATATTGGA 360

Query 61 CAATGGGCGGAAGCCTGATCCAGCAATACCGCGTGTGTGAAGAAGGCCTTCKGGTTGTAA 120

||||||||||||||||||||||||||||||||||||||||||||||||||| ||||||||

Sbjct 361 CAATGGGCGGAAGCCTGATCCAGCAATACCGCGTGTGTGAAGAAGGCCTTCGGGTTGTAA 420

Query 121 AGCACTTTTGTCAGGRAGAATGGCAGTTGAGGTTAATACCCTTAKCTGTTGATGTTACCT 180

||||||||||||||| |||||||||||||||||||||||||||| |||||||||||||||

Sbjct 421 AGCACTTTTGTCAGGGAGAATGGCAGTTGAGGTTAATACCCTTAGCTGTTGATGTTACCT 480

Query 181 GAAGAATAAGCACCGGCTAACTCCGTGCCAGCAGCCGCGGTAATACGGGGGGTGCAAGCG 240

||||||||||||||||||||||||||||||||||||||||||||||||||||||||||||

Sbjct 481 GAAGAATAAGCACCGGCTAACTCCGTGCCAGCAGCCGCGGTAATACGGGGGGTGCAAGCG 540

Query 241 TTAATCGGAATTACTGGGCGTAAAGGGCGCGTASGCGGTTACTTAAGTTAGATGTGAAAG 300

||||||||||||||||||||||||||||||||| ||||||||||||||||||||||||||

Sbjct 541 TTAATCGGAATTACTGGGCGTAAAGGGCGCGTAGGCGGTTACTTAAGTTAGATGTGAAAG 600

Query 301 CCCCGGGCTTAACCTGGGAATTGCATTTAATACTGGGTAACTAGAGTGTGGTAGAGAGTA 360

||||||||||||||||||||||||||||||||||||||||||||||||||||||||||||

Sbjct 601 CCCCGGGCTTAACCTGGGAATTGCATTTAATACTGGGTAACTAGAGTGTGGTAGAGAGTA 660

Query 361 GCGGAATTTCTGGTGTARCGGTGAAATGCRTAKATATCAGAASGAACACCAATGGCGAAS 420

||||||||||||||||| ||||||||||| || ||||||||| ||||||||||||||||

Sbjct 661 GCGGAATTTCTGGTGTAGCGGTGAAATGCGTAGATATCAGAAGGAACACCAATGGCGAAG 720

Query 421 GCAGCTGCTTGGRCCRACACTGACGCTGARGCGCGAAAGCGTGGGGAGCAAACAGGATTA 480

|||||||||||| || ||||||||||||| ||||||||||||||||||||||||||||||

Sbjct 721 GCAGCTGCTTGGGCCAACACTGACGCTGAGGCGCGAAAGCGTGGGGAGCAAACAGGATTA 780

Query 481 SATACCCTGGTAGTCCACGCTGTAAACGATGACAACTTATTGATGGGAGGGTTACCTTTC 540

|||||||||||||||||||||||||||||||||||||||||||||||||||||||||||

Sbjct 781 GATACCCTGGTAGTCCACGCTGTAAACGATGACAACTTATTGATGGGAGGGTTACCTTTC 840

Query 541 GTTGATGTAGCTAACGTGTTAAGTTGTCCGCCTGGGGAGTACGGTCGCAAGGCTGAAACT 600

||||||||||||||||||||||||||||||||||||||||||||||||||||||||||||

Sbjct 841 GTTGATGTAGCTAACGTGTTAAGTTGTCCGCCTGGGGAGTACGGTCGCAAGGCTGAAACT 900

Query 601 CAAAGGAATTGACGGGGACCCGCACAAGCGGTGGAGCATGTGGTTTAATTCGATGCAACG 660

||||||||||||||||||||||||||||||||||||||||||||||||||||||||||||

Sbjct 901 CAAAGGAATTGACGGGGACCCGCACAAGCGGTGGAGCATGTGGTTTAATTCGATGCAACG 960

Query 661 MGMAKAACCTTACCTGGTCTTGACATCTACAKAACTWTCCAGAGATGGATT-GTGCCTWC 719

| | |||||||||||||||||||||||||| |||| |||||||||||||| |||||| |

Sbjct 961 CGAAGAACCTTACCTGGTCTTGACATCTACAGAACTTTCCAGAGATGGATTGGTGCCTTC 1020

Query 720 WGGAACTGTAAGACAGGTGCTGCATGGCTGTCGTCAGCTCGTGWCRTGAGATGTAGGGTT 779

|||||||||||||||||||||||||||||||||||||||||| | |||||||| |||||

Sbjct 1021 GGGAACTGTAAGACAGGTGCTGCATGGCTGTCGTCAGCTCGTGTCGTGAGATGTTGGGTT 1080

Query 780 AAGTCC-GCAACGAAGCGCTATCCT-GTCCGTTATT 813

|||||| ||||||| |||| | ||| |||| |||||

Sbjct 1081 AAGTCCCGCAACGA-GCGCAACCCTTGTCC-TTATT 1114

1. **Sample_17: *K. pneumonia***


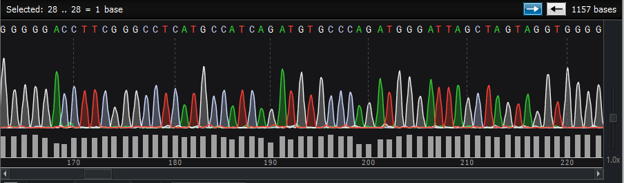


**Sanger di-deoxy sequencing chromatogram image of Sample_17: *K. pneumonia***

**Full sequence obtained from sanger sequencing**

CWMMGGGRMMRMMAWACAMATGCAAGTCGAGCGGTAGCACAGAGAGCTTGCTCTCGGGTGACGAGCGGCGGACGGGTGAGTAATGTCTGGGAAACTGCCTGATGGAGGGGGATAACTACTGGAAACGGTAGCTAATACCGCATAAYGTCGCAAGACCAAAGTGGGGGACCTTCGGGCCTCATGCCATCAGATGTGCCCAGATGGGATTAGCTAGTAGGTGGGGTAACGGCTCACCTAGGCGACGATCCCTAGCTGGTCTGAGAGGATGACCAGCCACACTGGAACTGAGACACGGTCCAGACTCCTACGGGAGGCAGCAGTGGGGAATATTGCACAATGGGCGCAAGCCTGATGCAGCCATGCCGCGTGTGTGAAGAAGGCCTTCGGGTTGTAAAGCACTTTCAGCGGGGAGGAAGGCGKTRAGGTTAATAACCTYGKCGATTGACGTTACCCGCAGAAGAAGCACCGGCTAACTCCGTGCCAGCAGCCGCGGTAATACGGAGGGTGCAAGCGTTAATCGGAATTACTGGGCGTAAAGCGCACGCAGGCGGTCTGTCAAGTCGGATGTGAAATCCCCGGGCTYAACCTGGGAACTGCATTCGAAACTGGCAGGCTAGAGTCTTGTAGAGGGGGGTAGAATTCCAGGTGTAGCGGTGAAATGCGTAGAGATCTGGAGGAATACCGGTGGCGAAGGCGGCCCCCTGGACAAAGACTGACGCTCAGGTGCGAAAGCGTGGGGAGCAAACAGGATTAGATACCCTGGTAGTCCACGCCGTAAACGATGTCGATTTGGAGGTTGTGCCCTTGAGGCGTGGCTTCCGGAGCTAACGCGTTAAATCGACCGCCTGGGGAGTACGGCCGCAAGGTTAAAACTCAAATGAATTGACGGGGGCCCGCACAAGCGGTGGAGCATGTGGTTTAATTCGATGCAACGCGAAGAACCTTACCTGGTCTTGACATCCACAGAACTTTCCAGAGATGGATTGGTGCCTTCGGGAACTGTGAGACAGGTGCTGCATGGCTGTCGTCAGCTCGTGTTGTGAATGTTGGGTTTAGTCCGCAACGAGCGCAACCTTATCCTTGTGCCAGCGGTTCGGGCCGGAACTCAAGAGACTGGCAGTGATAACCTGCTGGAAAGGGKTGGGGGAATTGAACCG

**Selected sequence for blast run (26-1021 bp)**

GTCGAGCGGTAGCACAGAGAGCTTGCTCTCGGGTGACGAGCGGCGGACGGGTGAGTAATGTCTGGGAAACTGCCTGATGGAGGGGGATAACTACTGGAAACGGTAGCTAATACCGCATAAYGTCGCAAGACCAAAGTGGGGGACCTTCGGGCCTCATGCCATCAGATGTGCCCAGATGGGATTAGCTAGTAGGTGGGGTAACGGCTCACCTAGGCGACGATCCCTAGCTGGTCTGAGAGGATGACCAGCCACACTGGAACTGAGACACGGTCCAGACTCCTACGGGAGGCAGCAGTGGGGAATATTGCACAATGGGCGCAAGCCTGATGCAGCCATGCCGCGTGTGTGAAGAAGGCCTTCGGGTTGTAAAGCACTTTCAGCGGGGAGGAAGGCGKTRAGGTTAATAACCTYGKCGATTGACGTTACCCGCAGAAGAAGCACCGGCTAACTCCGTGCCAGCAGCCGCGGTAATACGGAGGGTGCAAGCGTTAATCGGAATTACTGGGCGTAAAGCGCACGCAGGCGGTCTGTCAAGTCGGATGTGAAATCCCCGGGCTYAACCTGGGAACTGCATTCGAAACTGGCAGGCTAGAGTCTTGTAGAGGGGGGTAGAATTCCAGGTGTAGCGGTGAAATGCGTAGAGATCTGGAGGAATACCGGTGGCGAAGGCGGCCCCCTGGACAAAGACTGACGCTCAGGTGCGAAAGCGTGGGGAGCAAACAGGATTAGATACCCTGGTAGTCCACGCCGTAAACGATGTCGATTTGGAGGTTGTGCCCTTGAGGCGTGGCTTCCGGAGCTAACGCGTTAAATCGACCGCCTGGGGAGTACGGCCGCAAGGTTAAAACTCAAATGAATTGACGGGGGCCCGCACAAGCGGTGGAGCATGTGGTTTAATTCGATGCAACGCGAAGAACCTTACCTGGTCTTGACATCCACAGAACTTTCCAGAGATGGATTGGTGCCTTCGGGAACTGTGAGACAGGTGCTGCATGG

**Blast result of *K. pneumonia***

*Klebsiella pneumoniae* subsp. pneumoniae strain YS203 16S ribosomal RNA gene, partial sequence

Sequence ID: OP476397.1 Length: 1435

Range 1: 22 to 1017

Score:1818 bits (984), Expect:0.0, Identities:990/996 (99%), Gaps:0/996 (0%), Strand: Plus/Plus

Query 1 GTCGAGCGGTAGCACAGAGAGCTTGCTCTCGGGTGACGAGCGGCGGACGGGTGAGTAATG 60

||||||||||||||||||||||||||||||||||||||||||||||||||||||||||||

Sbjct 22 GTCGAGCGGTAGCACAGAGAGCTTGCTCTCGGGTGACGAGCGGCGGACGGGTGAGTAATG 81

Query 61 TCTGGGAAACTGCCTGATGGAGGGGGATAACTACTGGAAACGGTAGCTAATACCGCATAA 120

||||||||||||||||||||||||||||||||||||||||||||||||||||||||||||

Sbjct 82 TCTGGGAAACTGCCTGATGGAGGGGGATAACTACTGGAAACGGTAGCTAATACCGCATAA 141

Query 121 YGTCGCAAGACCAAAGTGGGGGACCTTCGGGCCTCATGCCATCAGATGTGCCCAGATGGG 180

|||||||||||||||||||||||||||||||||||||||||||||||||||||||||||

Sbjct 142 TGTCGCAAGACCAAAGTGGGGGACCTTCGGGCCTCATGCCATCAGATGTGCCCAGATGGG 201

Query 181 ATTAGCTAGTAGGTGGGGTAACGGCTCACCTAGGCGACGATCCCTAGCTGGTCTGAGAGG 240

||||||||||||||||||||||||||||||||||||||||||||||||||||||||||||

Sbjct 202 ATTAGCTAGTAGGTGGGGTAACGGCTCACCTAGGCGACGATCCCTAGCTGGTCTGAGAGG 261

Query 241 ATGACCAGCCACACTGGAACTGAGACACGGTCCAGACTCCTACGGGAGGCAGCAGTGGGG 300

||||||||||||||||||||||||||||||||||||||||||||||||||||||||||||

Sbjct 262 ATGACCAGCCACACTGGAACTGAGACACGGTCCAGACTCCTACGGGAGGCAGCAGTGGGG 321

Query 301 AATATTGCACAATGGGCGCAAGCCTGATGCAGCCATGCCGCGTGTGTGAAGAAGGCCTTC 360

||||||||||||||||||||||||||||||||||||||||||||||||||||||||||||

Sbjct 322 AATATTGCACAATGGGCGCAAGCCTGATGCAGCCATGCCGCGTGTGTGAAGAAGGCCTTC 381

Query 361 GGGTTGTAAAGCACTTTCAGCGGGGAGGAAGGCGKTRAGGTTAATAACCTYGKCGATTGA 420

|||||||||||||||||||||||||||||||||| | ||||||||||||| | |||||||

Sbjct 382 GGGTTGTAAAGCACTTTCAGCGGGGAGGAAGGCGGTGAGGTTAATAACCTTGTCGATTGA 441

Query 421 CGTTACCCGCAGAAGAAGCACCGGCTAACTCCGTGCCAGCAGCCGCGGTAATACGGAGGG 480

||||||||||||||||||||||||||||||||||||||||||||||||||||||||||||

Sbjct 442 CGTTACCCGCAGAAGAAGCACCGGCTAACTCCGTGCCAGCAGCCGCGGTAATACGGAGGG 501

Query 481 TGCAAGCGTTAATCGGAATTACTGGGCGTAAAGCGCACGCAGGCGGTCTGTCAAGTCGGA 540

||||||||||||||||||||||||||||||||||||||||||||||||||||||||||||

Sbjct 502 TGCAAGCGTTAATCGGAATTACTGGGCGTAAAGCGCACGCAGGCGGTCTGTCAAGTCGGA 561

Query 541 TGTGAAATCCCCGGGCTYAACCTGGGAACTGCATTCGAAACTGGCAGGCTAGAGTCTTGT 600

||||||||||||||||| ||||||||||||||||||||||||||||||||||||||||||

Sbjct 562 TGTGAAATCCCCGGGCTCAACCTGGGAACTGCATTCGAAACTGGCAGGCTAGAGTCTTGT 621

Query 601 AGAGGGGGGTAGAATTCCAGGTGTAGCGGTGAAATGCGTAGAGATCTGGAGGAATACCGG 660

||||||||||||||||||||||||||||||||||||||||||||||||||||||||||||

Sbjct 622 AGAGGGGGGTAGAATTCCAGGTGTAGCGGTGAAATGCGTAGAGATCTGGAGGAATACCGG 681

Query 661 TGGCGAAGGCGGCCCCCTGGACAAAGACTGACGCTCAGGTGCGAAAGCGTGGGGAGCAAA 720

||||||||||||||||||||||||||||||||||||||||||||||||||||||||||||

Sbjct 682 TGGCGAAGGCGGCCCCCTGGACAAAGACTGACGCTCAGGTGCGAAAGCGTGGGGAGCAAA 741

Query 721 CAGGATTAGATACCCTGGTAGTCCACGCCGTAAACGATGTCGATTTGGAGGTTGTGCCCT 780

||||||||||||||||||||||||||||||||||||||||||||||||||||||||||||

Sbjct 742 CAGGATTAGATACCCTGGTAGTCCACGCCGTAAACGATGTCGATTTGGAGGTTGTGCCCT 801

Query 781 TGAGGCGTGGCTTCCGGAGCTAACGCGTTAAATCGACCGCCTGGGGAGTACGGCCGCAAG 840

||||||||||||||||||||||||||||||||||||||||||||||||||||||||||||

Sbjct 802 TGAGGCGTGGCTTCCGGAGCTAACGCGTTAAATCGACCGCCTGGGGAGTACGGCCGCAAG 861

Query 841 GTTAAAACTCAAATGAATTGACGGGGGCCCGCACAAGCGGTGGAGCATGTGGTTTAATTC 900

||||||||||||||||||||||||||||||||||||||||||||||||||||||||||||

Sbjct 862 GTTAAAACTCAAATGAATTGACGGGGGCCCGCACAAGCGGTGGAGCATGTGGTTTAATTC 921

Query 901 GATGCAACGCGAAGAACCTTACCTGGTCTTGACATCCACAGAACTTTCCAGAGATGGATT 960

||||||||||||||||||||||||||||||||||||||||||||||||||||||||||||

Sbjct 922 GATGCAACGCGAAGAACCTTACCTGGTCTTGACATCCACAGAACTTTCCAGAGATGGATT 981

Query 961 GGTGCCTTCGGGAACTGTGAGACAGGTGCTGCATGG 996

||||||||||||||||||||||||||||||||||||

Sbjct 982 GGTGCCTTCGGGAACTGTGAGACAGGTGCTGCATGG 1017

1. **Sample_19: *E. coli* (Lactose negative)**


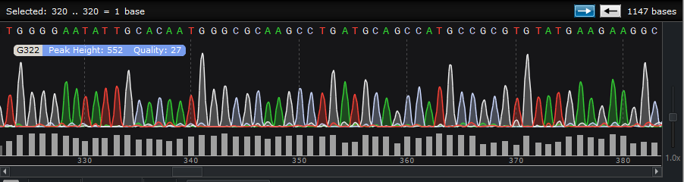


**Sanger di-deoxy sequencing chromatogram image of Sample_19: *E. coli* (Lactose negative)**

**Full sequence obtained from sanger sequencing**

AGSGGSAGCGWAMACATGCAASCWCGTAMSARKAAYARCGCYAGMAGCTTGCTKCTTYGCTGACGAGTGGCGGACGGGTGAGTAATGTCTGGGAAACTGCCTGATGGAGGGGGATAACTACTGGAAACGGTAGCTAATACCGCATAACGTCGCAAGACCAAAGAGGGGGACCTTCGGGCCTCTTGCCATCGGATGTGCCCAGATGGGATTAGCTAGTAGGTGGGGTAACGGCTCACCTAGGCGACGATCCCTAGCTGGTCTGAGAGGATGACCAGCCACACTGGAACTGAGACACGGTCCAGACTCCTACGGGAGGCAGCAGTGGGGAATATTGCACAATGGGCGCAAGCCTGATGCAGCCATGCCGCGTGTATGAAGAAGGCCTTCGGGTTGTAAAGTACTTTCAGCGGGGAGGAAGGGAGTAAAGTTAATACCTTTGCTCATTGACGTTACCCGCAGAAGAAGCACCGGCTAACTCCGTGCCAGCAGCCGCGGTAATACGGAGGGTGCAAGCGTTAATCGGAATTACTGGGCGTAAAGCGCACGCAGGCGGTTTGTTAAGTCAGATGTGAAATCCCCGGGCTCAACCTGGGAACTGCATCTGATACTGGCAAGCTTGAGTCTCGTAGAGGGGGGTAGAATTCCAGGTGTAGCGGTGAAATGCGTAGAGATCTGGAGGAATACCGGTGGCGAAGGCGGCCCCCTGGACGAAGACTGACGCTCAGGTGCGAAAGCGTGGGGAGCAAACAGGATTAGATACCCTGGTAGTCCACGCCGTAAACGATGTCGACTTGGAGGTTGTGCCCTTGAGGCGTGGCTTCCGGAGCTAACGCGTTAAGTCGACCGCCTGGGGAGTACGGCCGCAAGGTTAAAACTCAAATGAATTGACGGGGGCCCGCACAAGCGGTGGAGCATGTGGTTTAATTCGATGCAACGCGAAGAACCTTACCTGGTCTTGACATCCACAGAACTTTCCAGAGATGGATTGGTGCCTTCGGGAACTGTGAGACAGGTGCTGCATGGCTGTCGTCAGCTCGTGTTGTGAATGTTGGTTAAGTCCCGCACGAGCGCACCCTTATCCTTTGTTGCCAGCGTTCGCTGGTAACTCAAGGAGACTGCCAGTKGATAACCTGGGAGGAAGGTTGAG

**Selected sequence for blast run (59-1020 bp)**

GCTGACGAGTGGCGGACGGGTGAGTAATGTCTGGGAAACTGCCTGATGGAGGGGGATAACTACTGGAAACGGTAGCTAATACCGCATAACGTCGCAAGACCAAAGAGGGGGACCTTCGGGCCTCTTGCCATCGGATGTGCCCAGATGGGATTAGCTAGTAGGTGGGGTAACGGCTCACCTAGGCGACGATCCCTAGCTGGTCTGAGAGGATGACCAGCCACACTGGAACTGAGACACGGTCCAGACTCCTACGGGAGGCAGCAGTGGGGAATATTGCACAATGGGCGCAAGCCTGATGCAGCCATGCCGCGTGTATGAAGAAGGCCTTCGGGTTGTAAAGTACTTTCAGCGGGGAGGAAGGGAGTAAAGTTAATACCTTTGCTCATTGACGTTACCCGCAGAAGAAGCACCGGCTAACTCCGTGCCAGCAGCCGCGGTAATACGGAGGGTGCAAGCGTTAATCGGAATTACTGGGCGTAAAGCGCACGCAGGCGGTTTGTTAAGTCAGATGTGAAATCCCCGGGCTCAACCTGGGAACTGCATCTGATACTGGCAAGCTTGAGTCTCGTAGAGGGGGGTAGAATTCCAGGTGTAGCGGTGAAATGCGTAGAGATCTGGAGGAATACCGGTGGCGAAGGCGGCCCCCTGGACGAAGACTGACGCTCAGGTGCGAAAGCGTGGGGAGCAAACAGGATTAGATACCCTGGTAGTCCACGCCGTAAACGATGTCGACTTGGAGGTTGTGCCCTTGAGGCGTGGCTTCCGGAGCTAACGCGTTAAGTCGACCGCCTGGGGAGTACGGCCGCAAGGTTAAAACTCAAATGAATTGACGGGGGCCCGCACAAGCGGTGGAGCATGTGGTTTAATTCGATGCAACGCGAAGAACCTTACCTGGTCTTGACATCCACAGAACTTTCCAGAGATGGATTGGTGCCTTCGGGAACTGTGAGACAGGTGCTGCA

**Blast result of *E. coli***

>Escherichia coli strain 49 16S ribosomal RNA gene, partial sequence

Sequence ID: OP437652.1 Length: 1401

Range 1: 38 to 999

Score:1777 bits (962), Expect:0.0, Identities: 962/962 (100%), Gaps:0/962 (0%), Strand: Plus/Plus

Query 1 GCTGACGAGTGGCGGACGGGTGAGTAATGTCTGGGAAACTGCCTGATGGAGGGGGATAAC 60

||||||||||||||||||||||||||||||||||||||||||||||||||||||||||||

Sbjct 38 GCTGACGAGTGGCGGACGGGTGAGTAATGTCTGGGAAACTGCCTGATGGAGGGGGATAAC 97

Query 61 TACTGGAAACGGTAGCTAATACCGCATAACGTCGCAAGACCAAAGAGGGGGACCTTCGGG 120

||||||||||||||||||||||||||||||||||||||||||||||||||||||||||||

Sbjct 98 TACTGGAAACGGTAGCTAATACCGCATAACGTCGCAAGACCAAAGAGGGGGACCTTCGGG 157

Query 121 CCTCTTGCCATCGGATGTGCCCAGATGGGATTAGCTAGTAGGTGGGGTAACGGCTCACCT 180

||||||||||||||||||||||||||||||||||||||||||||||||||||||||||||

Sbjct 158 CCTCTTGCCATCGGATGTGCCCAGATGGGATTAGCTAGTAGGTGGGGTAACGGCTCACCT 217

Query 181 AGGCGACGATCCCTAGCTGGTCTGAGAGGATGACCAGCCACACTGGAACTGAGACACGGT 240

||||||||||||||||||||||||||||||||||||||||||||||||||||||||||||

Sbjct 218 AGGCGACGATCCCTAGCTGGTCTGAGAGGATGACCAGCCACACTGGAACTGAGACACGGT 277

Query 241 CCAGACTCCTACGGGAGGCAGCAGTGGGGAATATTGCACAATGGGCGCAAGCCTGATGCA 300

||||||||||||||||||||||||||||||||||||||||||||||||||||||||||||

Sbjct 278 CCAGACTCCTACGGGAGGCAGCAGTGGGGAATATTGCACAATGGGCGCAAGCCTGATGCA 337

Query 301 GCCATGCCGCGTGTATGAAGAAGGCCTTCGGGTTGTAAAGTACTTTCAGCGGGGAGGAAG 360

||||||||||||||||||||||||||||||||||||||||||||||||||||||||||||

Sbjct 338 GCCATGCCGCGTGTATGAAGAAGGCCTTCGGGTTGTAAAGTACTTTCAGCGGGGAGGAAG 397

Query 361 GGAGTAAAGTTAATACCTTTGCTCATTGACGTTACCCGCAGAAGAAGCACCGGCTAACTC 420

||||||||||||||||||||||||||||||||||||||||||||||||||||||||||||

Sbjct 398 GGAGTAAAGTTAATACCTTTGCTCATTGACGTTACCCGCAGAAGAAGCACCGGCTAACTC 457

Query 421 CGTGCCAGCAGCCGCGGTAATACGGAGGGTGCAAGCGTTAATCGGAATTACTGGGCGTAA 480

||||||||||||||||||||||||||||||||||||||||||||||||||||||||||||

Sbjct 458 CGTGCCAGCAGCCGCGGTAATACGGAGGGTGCAAGCGTTAATCGGAATTACTGGGCGTAA 517

Query 481 AGCGCACGCAGGCGGTTTGTTAAGTCAGATGTGAAATCCCCGGGCTCAACCTGGGAACTG 540

||||||||||||||||||||||||||||||||||||||||||||||||||||||||||||

Sbjct 518 AGCGCACGCAGGCGGTTTGTTAAGTCAGATGTGAAATCCCCGGGCTCAACCTGGGAACTG 577

Query 541 CATCTGATACTGGCAAGCTTGAGTCTCGTAGAGGGGGGTAGAATTCCAGGTGTAGCGGTG 600

||||||||||||||||||||||||||||||||||||||||||||||||||||||||||||

Sbjct 578 CATCTGATACTGGCAAGCTTGAGTCTCGTAGAGGGGGGTAGAATTCCAGGTGTAGCGGTG 637

Query 601 AAATGCGTAGAGATCTGGAGGAATACCGGTGGCGAAGGCGGCCCCCTGGACGAAGACTGA 660

||||||||||||||||||||||||||||||||||||||||||||||||||||||||||||

Sbjct 638 AAATGCGTAGAGATCTGGAGGAATACCGGTGGCGAAGGCGGCCCCCTGGACGAAGACTGA 697

Query 661 CGCTCAGGTGCGAAAGCGTGGGGAGCAAACAGGATTAGATACCCTGGTAGTCCACGCCGT 720

||||||||||||||||||||||||||||||||||||||||||||||||||||||||||||

Sbjct 698 CGCTCAGGTGCGAAAGCGTGGGGAGCAAACAGGATTAGATACCCTGGTAGTCCACGCCGT 757

Query 721 AAACGATGTCGACTTGGAGGTTGTGCCCTTGAGGCGTGGCTTCCGGAGCTAACGCGTTAA 780

||||||||||||||||||||||||||||||||||||||||||||||||||||||||||||

Sbjct 758 AAACGATGTCGACTTGGAGGTTGTGCCCTTGAGGCGTGGCTTCCGGAGCTAACGCGTTAA 817

Query 781 GTCGACCGCCTGGGGAGTACGGCCGCAAGGTTAAAACTCAAATGAATTGACGGGGGCCCG 840

||||||||||||||||||||||||||||||||||||||||||||||||||||||||||||

Sbjct 818 GTCGACCGCCTGGGGAGTACGGCCGCAAGGTTAAAACTCAAATGAATTGACGGGGGCCCG 877

Query 841 CACAAGCGGTGGAGCATGTGGTTTAATTCGATGCAACGCGAAGAACCTTACCTGGTCTTG 900

||||||||||||||||||||||||||||||||||||||||||||||||||||||||||||

Sbjct 878 CACAAGCGGTGGAGCATGTGGTTTAATTCGATGCAACGCGAAGAACCTTACCTGGTCTTG 937

Query 901 ACATCCACAGAACTTTCCAGAGATGGATTGGTGCCTTCGGGAACTGTGAGACAGGTGCTG 960

||||||||||||||||||||||||||||||||||||||||||||||||||||||||||||

Sbjct 938 ACATCCACAGAACTTTCCAGAGATGGATTGGTGCCTTCGGGAACTGTGAGACAGGTGCTG 997

Query 961 CA 962

||

Sbjct 998 CA 999

1. **Sample_20.2 (*S. enterica*)**


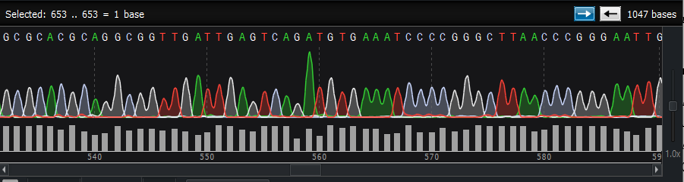


**Sanger di-deoxy sequencing chromatogram image of Sample_20.2 (*S. enterica*)**

**Full sequence obtained from sanger sequencing**

CTAGTCAGCTAMCATGCAGTCGTACAAGGTAACYARAAGCTTGCTTCTCTGCTGACGAGCGGCGGACGGGTGAGTAATGTATGGGGATCTGCCTGATGGAGGGGGATAACTACTGGAAACGGTAGCTAATACCGCATAATGTCTTCGGACCAAAGCGGGGGACCTCCGGGCCTCGCGCCATCAGATGAACCCATATGGGATTAGCTAGTAGGTGAGGTAACGGCTTACCTAGGCGACGATCCCTAGCTGGTCTGAGAGGATGATCAGCCACACTGGGACTGAGACACGGCCCAGACTCCTACGGGAGGCAGCAGTGGGGAATATTGCACAATGGGCGCAAGCCTGATGCAGCCATGCCGCGTGTATGAAGAAGGCCTTCGGGTTGTAAAGTACTTTCAGTCGGGAGGAAGGTGTCAAGGTTAATAACCTTGGCAATTGACGTTACCGACAGAAGAAGCACCGGCTAACTCCGTGCCAGCAGCCGCGGTAATACGGAGGGTGCAAGCGTTAATCGGAATTACTGGGCGTAAAGCGCACGCAGGCGGTTGATTGAGTCAGATGTGAAATCCCCGGGCTTAACCCGGGAATTGCATCTGATACTGGTCAGCTAGAGTCTTGTAGAGGGGGGTAGAATTCCATGTGTAGCGGTGAAATGCGTAGAGATGTGGAGGAATACCGGTGGCGAAGGCGGCCCCCTGGACAAAGACTGACGCTCAGGTGCGAAAGCGTGGGGAGCAAACAGGATTAGATACCCTGGTAGTCCACGCTGTAAACGATGTCGACTTGGAGGTTGTGCCCTTGAGGCGTGGCTTCCGGAGCTAACGCGTTAAGTCGACCGCCTGGGGGAGTACGGCCGCAAGGTTAAAACTCAAATGAATTGACGGGGGGGCCCGCACAAGCGGTGGAGCATGTGGTTTAATTCGATGCAACGCGAAGAACCTTACCTACTCTTGACATCCAGAGARCTTTWGCCAGAGATGGCATTTGGCTGCCTTTCGGGCACTTCTGAAGACAGTGCTGCATGGCCTGGTCGATCAGGCTCGATGT

**Selected sequence for blast run (37-840 bp)**

AAGCTTGCTTCTCTGCTGACGAGCGGCGGACGGGTGAGTAATGTATGGGGATCTGCCTGATGGAGGGGGATAACTACTGGAAACGGTAGCTAATACCGCATAATGTCTTCGGACCAAAGCGGGGGACCTCCGGGCCTCGCGCCATCAGATGAACCCATATGGGATTAGCTAGTAGGTGAGGTAACGGCTTACCTAGGCGACGATCCCTAGCTGGTCTGAGAGGATGATCAGCCACACTGGGACTGAGACACGGCCCAGACTCCTACGGGAGGCAGCAGTGGGGAATATTGCACAATGGGCGCAAGCCTGATGCAGCCATGCCGCGTGTATGAAGAAGGCCTTCGGGTTGTAAAGTACTTTCAGTCGGGAGGAAGGTGTCAAGGTTAATAACCTTGGCAATTGACGTTACCGACAGAAGAAGCACCGGCTAACTCCGTGCCAGCAGCCGCGGTAATACGGAGGGTGCAAGCGTTAATCGGAATTACTGGGCGTAAAGCGCACGCAGGCGGTTGATTGAGTCAGATGTGAAATCCCCGGGCTTAACCCGGGAATTGCATCTGATACTGGTCAGCTAGAGTCTTGTAGAGGGGGGTAGAATTCCATGTGTAGCGGTGAAATGCGTAGAGATGTGGAGGAATACCGGTGGCGAAGGCGGCCCCCTGGACAAAGACTGACGCTCAGGTGCGAAAGCGTGGGGAGCAAACAGGATTAGATACCCTGGTAGTCCACGCTGTAAACGATGTCGACTTGGAGGTTGTGCCCTTGAGGCGTGGCTTCCGGAGCTAACGCGTTAAGTCGACCGCCTGGGGGAGTACGGCCGCAAGGTTAAAACTCAAATGA

**Blast result *S. enterica***

*Salmonella enterica* subsp. enterica serovar Newport strain CP012598.1 16S ribosomal RNA gene, partial sequence

Sequence ID: MH356687.1 Length: 1069

Range 1: 22 to 860

Score:1544 bits (836), Expect: 0.0, Identities: 839/840 (99%), Gaps: 1/840 (0%), Strand: Plus/Plus

Query 1 AAGCTTGCTTCTCTGCTGACGAGCGGCGGACGGGTGAGTAATGTATGGGGATCTGCCTGA 60

||||||||||||||||||||||||||||||||||||||||||||||||||||||||||||

Sbjct 22 AAGCTTGCTTCTCTGCTGACGAGCGGCGGACGGGTGAGTAATGTATGGGGATCTGCCTGA 81

Query 61 TGGAGGGGGATAACTACTGGAAACGGTAGCTAATACCGCATAATGTCTTCGGACCAAAGC 120

||||||||||||||||||||||||||||||||||||||||||||||||||||||||||||

Sbjct 82 TGGAGGGGGATAACTACTGGAAACGGTAGCTAATACCGCATAATGTCTTCGGACCAAAGC 141

Query 121 GGGGGACCTCCGGGCCTCGCGCCATCAGATGAACCCATATGGGATTAGCTAGTAGGTGAG 180

||||||||||||||||||||||||||||||||||||||||||||||||||||||||||||

Sbjct 142 GGGGGACCTCCGGGCCTCGCGCCATCAGATGAACCCATATGGGATTAGCTAGTAGGTGAG 201

Query 181 GTAACGGCTTACCTAGGCGACGATCCCTAGCTGGTCTGAGAGGATGATCAGCCACACTGG 240

||||||||||||||||||||||||||||||||||||||||||||||||||||||||||||

Sbjct 202 GTAACGGCTTACCTAGGCGACGATCCCTAGCTGGTCTGAGAGGATGATCAGCCACACTGG 261

Query 241 GACTGAGACACGGCCCAGACTCCTACGGGAGGCAGCAGTGGGGAATATTGCACAATGGGC 300

||||||||||||||||||||||||||||||||||||||||||||||||||||||||||||

Sbjct 262 GACTGAGACACGGCCCAGACTCCTACGGGAGGCAGCAGTGGGGAATATTGCACAATGGGC 321

Query 301 GCAAGCCTGATGCAGCCATGCCGCGTGTATGAAGAAGGCCTTCGGGTTGTAAAGTACTTT 360

||||||||||||||||||||||||||||||||||||||||||||||||||||||||||||

Sbjct 322 GCAAGCCTGATGCAGCCATGCCGCGTGTATGAAGAAGGCCTTCGGGTTGTAAAGTACTTT 381

Query 361 CAGTCGGGAGGAAGGTGTCAAGGTTAATAACCTTGGCAATTGACGTTACCGACAGAAGAA 420

||||||||||||||||||||||||||||||||||||||||||||||||||||||||||||

Sbjct 382 CAGTCGGGAGGAAGGTGTCAAGGTTAATAACCTTGGCAATTGACGTTACCGACAGAAGAA 441

Query 421 GCACCGGCTAACTCCGTGCCAGCAGCCGCGGTAATACGGAGGGTGCAAGCGTTAATCGGA 480

||||||||||||||||||||||||||||||||||||||||||||||||||||||||||||

Sbjct 442 GCACCGGCTAACTCCGTGCCAGCAGCCGCGGTAATACGGAGGGTGCAAGCGTTAATCGGA 501

Query 481 ATTACTGGGCGTAAAGCGCACGCAGGCGGTTGATTGAGTCAGATGTGAAATCCCCGGGCT 540

||||||||||||||||||||||||||||||||||||||||||||||||||||||||||||

Sbjct 502 ATTACTGGGCGTAAAGCGCACGCAGGCGGTTGATTGAGTCAGATGTGAAATCCCCGGGCT 561

Query 541 TAACCCGGGAATTGCATCTGATACTGGTCAGCTAGAGTCTTGTAGAGGGGGGTAGAATTC 600

||||||||||||||||||||||||||||||||||||||||||||||||||||||||||||

Sbjct 562 TAACCCGGGAATTGCATCTGATACTGGTCAGCTAGAGTCTTGTAGAGGGGGGTAGAATTC 621

Query 601 CATGTGTAGCGGTGAAATGCGTAGAGATGTGGAGGAATACCGGTGGCGAAGGCGGCCCCC 660

||||||||||||||||||||||||||||||||||||||||||||||||||||||||||||

Sbjct 622 CATGTGTAGCGGTGAAATGCGTAGAGATGTGGAGGAATACCGGTGGCGAAGGCGGCCCCC 681

Query 661 TGGACAAAGACTGACGCTCAGGTGCGAAAGCGTGGGGAGCAAACAGGATTAGATACCCTG 720

||||||||||||||||||||||||||||||||||||||||||||||||||||||||||||

Sbjct 682 TGGACAAAGACTGACGCTCAGGTGCGAAAGCGTGGGGAGCAAACAGGATTAGATACCCTG 741

Query 721 GTAGTCCACGCTGTAAACGATGTCGACTTGGAGGTTGTGCCCTTGAGGCGTGGCTTCCGG 780

||||||||||||||||||||||||||||||||||||||||||||||||||||||||||||

Sbjct 742 GTAGTCCACGCTGTAAACGATGTCGACTTGGAGGTTGTGCCCTTGAGGCGTGGCTTCCGG 801

Query 781 AGCTAACGCGTTAAGTCGACCGCCTGGGGGAGTACGGCCGCAAGGTTAAAACTCAAATGA 840

||||||||||||||||||||||||| ||||||||||||||||||||||||||||||||||

Sbjct 802 AGCTAACGCGTTAAGTCGACCGCCT-GGGGAGTACGGCCGCAAGGTTAAAACTCAAATGA 860

1. **Sample_16.2: *P.* *penneri***


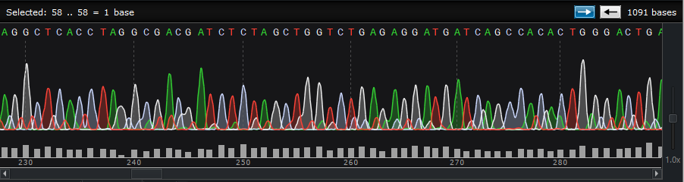


**Sanger di-deoxy sequencing chromatogram image of Sample_16.2: *P.* *penneri***

**Full sequence obtained from sanger sequencing**

CTGCGGSRGGCGTACACATGCAGTCGWGCGRKGAYARCMAGAAAAGCTTGCTTTCTTGCTGACGAGCGGCGGAGGGGKGRRGARTGTATGGGGATCKGCCCGATAGAGGGGGATAAMTACTGGAAACGGWGGCTAATACCGCATGACGTCTASGGACCAAAGCAGGGGCTCTTCGGACCTTGCSCTATCRGATGAACCCATATGGGAKTAGCTAGTAGGTGAGGTAAAGGCTCACCTAGGCGACGATCTCTAGCTGGTCTGAGAGGATGATCAGCCACACTGGGACTGAGACACGGCCCAGACTCCTACGGGAGGCAGCAGTGGGGAATATTGCACAATGGGCGCAAGCCTGATGCAGCCATGCCGCGTGTATGAAGAAGGCCTTAGGGTTGTAAAGTACTTTCASCGGGGAGGAAGGTGATAAAGTTAATACCTTTRTCAATTGACGTTACCCGCAGAASAAGCACCGGCTAACTCCGTGCCRGCAGCCGCGGTAATACGGAGGGTGCAAGCGTTAATCGGAATTACTGGGCGTAAAGCGCACGCRGGCGGTCAATTAAGTCAKATGTGAAAGCCCCGAGCTTAACTTGGGAATTGCATCTGAAACTGGTTGGCTAGAGTCTTGTARAGGGGGGTAGAATTCCATGTGTAGCGGTGAAATGCKTAGAGATGTGGAGGAATACCGGTGGCGAAGGCGGYCCCCTGGACAAAGACTGACGCTCAKGTGCGAAAGCGTGKGGAGCAAACAGGATTAKATACCCTGGTAKTCCACGCTGTATACGATGTCKATTTAGAGGTTGTGGTCTTGAACCGYGGCTTCTGGAGMTAACGCGTTAAATCGACCGCMTGGGRGAGTACGGCCGCAAGGTTAAAAACTTCAAATGAATTGACRGGGGGCCCGCACAAGCGGTGGAKCATGTGGTTTAATTCGATGCAACGCGAAGAAACCTTACCTTACTCTTGACATCCAGCGRCATCCTTTAGAGATAGCAKGCAGTTGYCCTTTCGGGAAACGCTGAGACTAGGRTGCTGYAATGGTCTGATTCRTCAGTCCTCCGCTYGCYTTGSTGGAAAATTGGTATCTGKGTGTG

**Selected sequence for blast run (118-1043 bp)**

TACTGGAAACGGWGGCTAATACCGCATGACGTCTASGGACCAAAGCAGGGGCTCTTCGGACCTTGCSCTATCRGATGAACCCATATGGGAKTAGCTAGTAGGTGAGGTAAAGGCTCACCTAGGCGACGATCTCTAGCTGGTCTGAGAGGATGATCAGCCACACTGGGACTGAGACACGGCCCAGACTCCTACGGGAGGCAGCAGTGGGGAATATTGCACAATGGGCGCAAGCCTGATGCAGCCATGCCGCGTGTATGAAGAAGGCCTTAGGGTTGTAAAGTACTTTCASCGGGGAGGAAGGTGATAAAGTTAATACCTTTRTCAATTGACGTTACCCGCAGAASAAGCACCGGCTAACTCCGTGCCRGCAGCCGCGGTAATACGGAGGGTGCAAGCGTTAATCGGAATTACTGGGCGTAAAGCGCACGCRGGCGGTCAATTAAGTCAKATGTGAAAGCCCCGAGCTTAACTTGGGAATTGCATCTGAAACTGGTTGGCTAGAGTCTTGTARAGGGGGGTAGAATTCCATGTGTAGCGGTGAAATGCKTAGAGATGTGGAGGAATACCGGTGGCGAAGGCGGYCCCCTGGACAAAGACTGACGCTCAKGTGCGAAAGCGTGKGGAGCAAACAGGATTAKATACCCTGGTAKTCCACGCTGTATACGATGTCKATTTAGAGGTTGTGGTCTTGAACCGYGGCTTCTGGAGMTAACGCGTTAAATCGACCGCMTGGGRGAGTACGGCCGCAAGGTTAAAAACTTCAAATGAATTGACRGGGGGCCCGCACAAGCGGTGGAKCATGTGGTTTAATTCGATGCAACGCGAAGAAACCTTACCTTACTCTTGACATCCAGCGRCATCCTTTAGAGATAGCAKGCAGTTGYCCTTTCGGGAAACGCTGAGACTAGGRTGCTGYAATGGTCTGA

**Blast result of *P. penneri***

*Proteus penneri* strain sar15 16S ribosomal RNA gene, partial sequence

Sequence ID: MT557004.1 Length: 1330

Range 1: 96 to 1003

Score:1496 bits (810), Expect:0.0, Identities: 881/925 (95%), Gaps:17/925 (1%), Strand: Plus/Plus

Query 1 TACTGGAAACGGWGGCTAATACCGCATGACGTCTASGGACCAAAGCAGGGGCTCTTCGGA 60

|||||||||||| |||||||||||||||||||||| ||||||||||||||||||||||||

Sbjct 96 TACTGGAAACGGTGGCTAATACCGCATGACGTCTACGGACCAAAGCAGGGGCTCTTCGGA 155

Query 61 CCTTGCSCTATCRGATGAACCCATATGGGAKTAGCTAGTAGGTGAGGTAAAGGCTCACCT 120

|||||| ||||| ||||||||||||||||| |||||||||||||||||||||||||||||

Sbjct 156 CCTTGCGCTATCGGATGAACCCATATGGGATTAGCTAGTAGGTGAGGTAAAGGCTCACCT 215

Query 121 AGGCGACGATCTCTAGCTGGTCTGAGAGGATGATCAGCCACACTGGGACTGAGACACGGC 180

||||||||||||||||||||||||||||||||||||||||||||||||||||||||||||

Sbjct 216 AGGCGACGATCTCTAGCTGGTCTGAGAGGATGATCAGCCACACTGGGACTGAGACACGGC 275

Query 181 CCAGACTCCTACGGGAGGCAGCAGTGGGGAATATTGCACAATGGGCGCAAGCCTGATGCA 240

||||||||||||||||||||||||||||||||||||||||||||||||||||||||||||

Sbjct 276 CCAGACTCCTACGGGAGGCAGCAGTGGGGAATATTGCACAATGGGCGCAAGCCTGATGCA 335

Query 241 GCCATGCCGCGTGTATGAAGAAGGCCTTAGGGTTGTAAAGTACTTTCASCGGGGAGGAAG 300

|||||||||||||||||||||||||||||||||||||||||||||||| |||||||||||

Sbjct 336 GCCATGCCGCGTGTATGAAGAAGGCCTTAGGGTTGTAAAGTACTTTCAGCGGGGAGGAAG 395

Query 301 GTGATAAAGTTAATACCTTTRTCAATTGACGTTACCCGCAGAASAAGCACCGGCTAACTC 360

|||||||||||||||||||| |||||||||||||||||||||| ||||||||||||||||

Sbjct 396 GTGATAAAGTTAATACCTTTATCAATTGACGTTACCCGCAGAAGAAGCACCGGCTAACTC 455

Query 361 CGTGCCRGCAGCCGCGGTAATACGGAGGGTGCAAGCGTTAATCGGAATTACTGGGCGTAA 420

|||||| |||||||||||||||||||||||||||||||||||||||||||||||||||||

Sbjct 456 CGTGCCAGCAGCCGCGGTAATACGGAGGGTGCAAGCGTTAATCGGAATTACTGGGCGTAA 515

Query 421 AGCGCACGCRGGCGGTCAATTAAGTCAKATGTGAAAGCCCCGAGCTTAACTTGGGAATTG 480

||||||||| ||||||||||||||||| ||||||||||||||||||||||||||||||||

Sbjct 516 AGCGCACGCAGGCGGTCAATTAAGTCAGATGTGAAAGCCCCGAGCTTAACTTGGGAATTG 575

Query 481 CATCTGAAACTGGTTGGCTAGAGTCTTGTARAGGGGGGTAGAATTCCATGTGTAGCGGTG 540

|||||||||||||||||||||||||||||| |||||||||||||||||||||||||||||

Sbjct 576 CATCTGAAACTGGTTGGCTAGAGTCTTGTAGAGGGGGGTAGAATTCCATGTGTAGCGGTG 635

Query 541 AAATGCKTAGAGATGTGGAGGAATACCGGTGGCGAAGGCGGYCCCCTGGACAAAGACTGA 600

|||||| |||||||||||||||||||||||||||||||||| ||||||||||||||||||

Sbjct 636 AAATGCGTAGAGATGTGGAGGAATACCGGTGGCGAAGGCGGCCCCCTGGACAAAGACTGA 695

Query 601 CGCTCAKGTGCGAAAGCGTGKGGAGCAAACAGGATTAKATACCCTGGTAKTCCACGCTGT 660

|||||| ||||||||||||| |||||||||||||||| ||||||||||| ||||||||||

Sbjct 696 CGCTCAGGTGCGAAAGCGTGGGGAGCAAACAGGATTAGATACCCTGGTAGTCCACGCTGT 755

Query 661 ATACGATGTCKATTTAGAGGTTGTGGTCTTGAACCGYGGCTTCTGGAGMTAACGCGTTAA 720

| |||||||| ||||||||||||||||||||||||| ||||||||||| |||||||||||

Sbjct 756 AAACGATGTCGATTTAGAGGTTGTGGTCTTGAACCGTGGCTTCTGGAGCTAACGCGTTAA 815

Query 721 ATCGACCGCMTGGGRGAGTACGGCCGCAAGGTTAAAAACTTCAAATGAATTGACRGGGGG 780

||||||||| |||| |||||||||||||||||||||| | |||||||||||||| |||||

Sbjct 816 ATCGACCGCCTGGG-GAGTACGGCCGCAAGGTTAAAA-C-TCAAATGAATTGAC-GGGGG 871

Query 781 CCCGCACAAGCGGTGGAKCATGTGGTTTAATTCGATGCAACGCGAAGAAACCTTACCTTA 840

||||||||||||||||| ||||||||||||||||||||||||||||||| ||||||| ||

Sbjct 872 CCCGCACAAGCGGTGGAGCATGTGGTTTAATTCGATGCAACGCGAAGAA-CCTTACC-TA 929

Query 841 CTCTTGACATCCAGCGRCATCCTTTAGAGATAGCAKGCAGTTGYCCTTTCGGGAAACGCT 900

|||||||||||||||| ||||||||||||||| | | ||| | |||| |||||| ||||

Sbjct 930 CTCTTGACATCCAGCGG-ATCCTTTAGAGATAG-AGG-AGT-G-CCTT-CGGGAA-CGCT 982

Query 901 GAGACTAGGRTGCTGYAATGGTCTG 925

||||| ||| ||||| | ||| |||

Sbjct 983 GAGAC-AGG-TGCTGCA-TGG-CTG 1003

1. **Sample_14.2: *M. morganii***


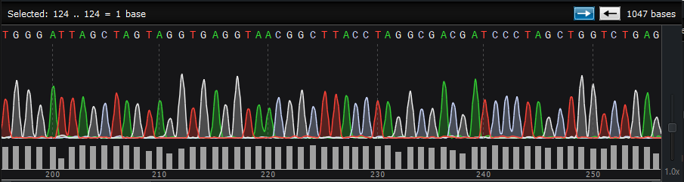


**Sanger di-deoxy sequencing chromatogram image of Sample_14.2: *M. morganii***

**Full sequence obtained from sanger sequencing**

CTAGTCAGCTAMCATGCAGTCGTACAAGGTAACYARAAGCTTGCTTCTCTGCTGACGAGCGGCGGACGGGTGAGTAATGTATGGGGATCTGCCTGATGGCGGGGGATAACTACTGGAAACGGTAGCTAATACCGCATAATGTCTTCGGACCAAAGCGGGGGACCTCCGGGCCTCGCGCCATCAGATGAACCCATATGGGATTAGCTAGTAGGTGAGGTAACGGCTTACCTAGGCGACGATCCCTAGCTGGTCTGAGAGGATGATCAGCCACACTGGGACTGAGACACGGCCCAGACTCCTACGGGAGGCAGCAGTGGGGAATATTGCACAATGGGCGCAAGCCTGATGCAGCCATGCCGCGTGTATGAAGAAGGCCTTCGGGTTGTAAAGTACTTTCAGTCGGGAGGAAGGTGTCAAGGTTAATAACCTTGGCAATTGACGTTACCGACAGAAGAAGCACCGGCTAACTCCGTGCCAGCAGCCGCGGTAATACGGAGGGTGCAAGCGTTAATCGGAATTACTGGGCGTAAAGCGCACGCAGGCGGTTGATTGAGTCAGATGTGAAATCCCCGGGCTTAACCCGGGAATTGCATCTGATACTGGTCAGCTAGAGTCTTGTAGAGGGGGGTAGAATTCCATGTGTAGCGGTGAAATGCGTAGAGATGTGGAGGAATACCGGTGGCGAAGGCGGCCCCCTGGACAAAGACTGACGCTCAGGTGCGAAAGCGTGGGGAGCAAACAGGATTAGATACCCTGGTAGTCCACGCTGTAAACGATGTCGACTTGGAGGTTGTGCCCTTGAGGCGTGGCTTCCGGAGCTAACGCGTTAAGTCGACCGCCTGGGGGAGTACGGCCGCAAGGTTAAAACTCAAATGAATTGACGGGGGGGCCCGCACAAGCGGTGGAGCATGTGGTTTAATTCGATGCAACGCGAAGAACCTTACCTACTCTTGACATCCAGAGARCTTTWGCCAGAGATGGCATTTGGCTGCCTTTCGGGCACTTCTGAAGACAGTGCTGCATGGCCTGGTCGATCAGGCTCGATGT

**Selected sequence for blast run (37-996 bp)**

AAGCTTGCTTCTCTGCTGACGAGCGGCGGACGGGTGAGTAATGTATGGGGATCTGCCTGATGGCGGGGGATAACTACTGGAAACGGTAGCTAATACCGCATAATGTCTTCGGACCAAAGCGGGGGACCTCCGGGCCTCGCGCCATCAGATGAACCCATATGGGATTAGCTAGTAGGTGAGGTAACGGCTTACCTAGGCGACGATCCCTAGCTGGTCTGAGAGGATGATCAGCCACACTGGGACTGAGACACGGCCCAGACTCCTACGGGAGGCAGCAGTGGGGAATATTGCACAATGGGCGCAAGCCTGATGCAGCCATGCCGCGTGTATGAAGAAGGCCTTCGGGTTGTAAAGTACTTTCAGTCGGGAGGAAGGTGTCAAGGTTAATAACCTTGGCAATTGACGTTACCGACAGAAGAAGCACCGGCTAACTCCGTGCCAGCAGCCGCGGTAATACGGAGGGTGCAAGCGTTAATCGGAATTACTGGGCGTAAAGCGCACGCAGGCGGTTGATTGAGTCAGATGTGAAATCCCCGGGCTTAACCCGGGAATTGCATCTGATACTGGTCAGCTAGAGTCTTGTAGAGGGGGGTAGAATTCCATGTGTAGCGGTGAAATGCGTAGAGATGTGGAGGAATACCGGTGGCGAAGGCGGCCCCCTGGACAAAGACTGACGCTCAGGTGCGAAAGCGTGGGGAGCAAACAGGATTAGATACCCTGGTAGTCCACGCTGTAAACGATGTCGACTTGGAGGTTGTGCCCTTGAGGCGTGGCTTCCGGAGCTAACGCGTTAAGTCGACCGCCTGGGGGAGTACGGCCGCAAGGTTAAAACTCAAATGAATTGACGGGGGGGCCCGCACAAGCGGTGGAGCATGTGGTTTAATTCGATGCAACGCGAAGAACCTTACCTACTCTTGACATCCAGAGARCTTTWGCCAGAGATGGCATTTGGCTGCCTTT

**Blast result of *M. morganii***

*Morganella morganii* strain ab1 16S ribosomal RNA gene, partial sequence

Sequence ID: MN744697.1 Length: 1464

Range 1: 51 to 1001

Score:1716 bits (929), Expect: 0.0, Identities:950/959(99%), Gaps:8/959 (0%), Strand: Plus/Plus

Query 1 AAGCTTGCTTCTCTGCTGACGAGCGGCGGACGGGTGAGTAATGTATGGGGATCTGCCTGA 60

||||||||||||||||||||||||||||||||||||||||||||||||||||||||||||

Sbjct 51 AAGCTTGCTTCTCTGCTGACGAGCGGCGGACGGGTGAGTAATGTATGGGGATCTGCCTGA 110

Query 61 TGGCGGGGGATAACTACTGGAAACGGTAGCTAATACCGCATAATGTCTTCGGACCAAAGC 120

||||||||||||||||||||||||||||||||||||||||||||||||||||||||||||

Sbjct 111 TGGCGGGGGATAACTACTGGAAACGGTAGCTAATACCGCATAATGTCTTCGGACCAAAGC 170

Query 121 GGGGGACCTCCGGGCCTCGCGCCATCAGATGAACCCATATGGGATTAGCTAGTAGGTGAG 180

||||||||||||||||||||||||||||||||||||||||||||||||||||||||||||

Sbjct 171 GGGGGACCTCCGGGCCTCGCGCCATCAGATGAACCCATATGGGATTAGCTAGTAGGTGAG 230

Query 181 GTAACGGCTTACCTAGGCGACGATCCCTAGCTGGTCTGAGAGGATGATCAGCCACACTGG 240

||||||||||||||||||||||||||||||||||||||||||||||||||||||||||||

Sbjct 231 GTAACGGCTTACCTAGGCGACGATCCCTAGCTGGTCTGAGAGGATGATCAGCCACACTGG 290

Query 241 GACTGAGACACGGCCCAGACTCCTACGGGAGGCAGCAGTGGGGAATATTGCACAATGGGC 300

||||||||||||||||||||||||||||||||||||||||||||||||||||||||||||

Sbjct 291 GACTGAGACACGGCCCAGACTCCTACGGGAGGCAGCAGTGGGGAATATTGCACAATGGGC 350

Query 301 GCAAGCCTGATGCAGCCATGCCGCGTGTATGAAGAAGGCCTTCGGGTTGTAAAGTACTTT 360

||||||||||||||||||||||||||||||||||||||||||||||||||||||||||||

Sbjct 351 GCAAGCCTGATGCAGCCATGCCGCGTGTATGAAGAAGGCCTTCGGGTTGTAAAGTACTTT 410

Query 361 CAGTCGGGAGGAAGGTGTCAAGGTTAATAACCTTGGCAATTGACGTTACCGACAGAAGAA 420

||||||||||||||||||||||||||||||||||||||||||||||||||||||||||||

Sbjct 411 CAGTCGGGAGGAAGGTGTCAAGGTTAATAACCTTGGCAATTGACGTTACCGACAGAAGAA 470

Query 421 GCACCGGCTAACTCCGTGCCAGCAGCCGCGGTAATACGGAGGGTGCAAGCGTTAATCGGA 480

||||||||||||||||||||||||||||||||||||||||||||||||||||||||||||

Sbjct 471 GCACCGGCTAACTCCGTGCCAGCAGCCGCGGTAATACGGAGGGTGCAAGCGTTAATCGGA 530

Query 481 ATTACTGGGCGTAAAGCGCACGCAGGCGGTTGATTGAGTCAGATGTGAAATCCCCGGGCT 540

||||||||||||||||||||||||||||||||||||||||||||||||||||||||||||

Sbjct 531 ATTACTGGGCGTAAAGCGCACGCAGGCGGTTGATTGAGTCAGATGTGAAATCCCCGGGCT 590

Query 541 TAACCCGGGAATTGCATCTGATACTGGTCAGCTAGAGTCTTGTAGAGGGGGGTAGAATTC 600

||||||||||||||||||||||||||||||||||||||||||||||||||||||||||||

Sbjct 591 TAACCCGGGAATTGCATCTGATACTGGTCAGCTAGAGTCTTGTAGAGGGGGGTAGAATTC 650

Query 601 CATGTGTAGCGGTGAAATGCGTAGAGATGTGGAGGAATACCGGTGGCGAAGGCGGCCCCC 660

||||||||||||||||||||||||||||||||||||||||||||||||||||||||||||

Sbjct 651 CATGTGTAGCGGTGAAATGCGTAGAGATGTGGAGGAATACCGGTGGCGAAGGCGGCCCCC 710

Query 661 TGGACAAAGACTGACGCTCAGGTGCGAAAGCGTGGGGAGCAAACAGGATTAGATACCCTG 720

||||||||||||||||||||||||||||||||||||||||||||||||||||||||||||

Sbjct 711 TGGACAAAGACTGACGCTCAGGTGCGAAAGCGTGGGGAGCAAACAGGATTAGATACCCTG 770

Query 721 GTAGTCCACGCTGTAAACGATGTCGACTTGGAGGTTGTGCCCTTGAGGCGTGGCTTCCGG 780

||||||||||||||||||||||||||||||||||||||||||||||||||||||||||||

Sbjct 771 GTAGTCCACGCTGTAAACGATGTCGACTTGGAGGTTGTGCCCTTGAGGCGTGGCTTCCGG 830

Query 781 AGCTAACGCGTTAAGTCGACCGCCTGGGGGAGTACGGCCGCAAGGTTAAAACTCAAATGA 840

||||||||||||||||||||||||| ||||||||||||||||||||||||||||||||||

Sbjct 831 AGCTAACGCGTTAAGTCGACCGCCT-GGGGAGTACGGCCGCAAGGTTAAAACTCAAATGA 889

Query 841 ATTGACGGGGGGGCCCGCACAAGCGGTGGAGCATGTGGTTTAATTCGATGCAACGCGAAG 900

|||||| ||||||||||||||||||||||||||||||||||||||||||||||||||||

Sbjct 890 ATTGAC--GGGGGCCCGCACAAGCGGTGGAGCATGTGGTTTAATTCGATGCAACGCGAAG 947

Query 901 AACCTTACCTACTCTTGACATCCAGAGARCTTTWGCCAGAGATGGCATTTGGCTGCCTT 959

|||||||||||||||||||||||||||| |||| |||||||||| ||| || ||||||

Sbjct 948 AACCTTACCTACTCTTGACATCCAGAGAACTTT--CCAGAGATGG-ATT-GG-TGCCTT 1001

1. **Sample_29: *E. coli* (lactose positive)**


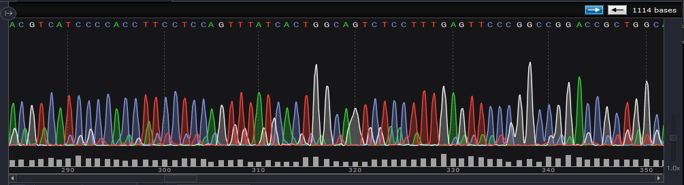


**Sanger di-deoxy sequencing chromatogram image of Sample_29: *E. coli* (lactose positive)**

**Full sequence obtained from sanger sequencing**

CKACGCAGCTACAATGCAGTCGTAASARGAATAKKTTAAAGCTTGCTKCTTTGCTGACGAGTGGCGGWCGGGTGAGTAATGTCTGGGAAACTGCCTGATGGAGGGGGATAACTACTGGAAACGGTAGCTAATACCGCATAACGTCGCRRGACCAAAGAGGGGGACCTTCGGGCCTCTTGCCATCGGATGTGCCCAGATGGGATTAGCTAGTAGGTGGGGTAACGGCTCACCTAGGCGACGATCCCTAGCTGGTCTGAGAGGATGACCRGCCACRCTGGAACTGAGACACGGTCCASACTCCTACGGGAGGCAGCAGTGGGGAATATTGCACAATGGGCGCAAGCCTGATGCASCCATGCCGCGKGTATGAAKAAGGCCTTCGGGTTGTAAAGTACTTTCAGCGGGGAGGAAGGGAGTAAAGTTAATACCTTTGCTCATTGACGTTACCCGCASAAKAAGCACCGGCTAACTCCGTGCCAGCAGCCGCGGTAATACGGAGGGTGCAAGCGTTAATCGGAATTACTGGGCGTAAAGCGCACGSAGGCGGTTTGTTAAGTCAGATGTGAAATCCCCGGGCTCAACCTGGGAACTGCATCTGATACTGGCAAGCTTGAGTCTCGTAGAGGGGGGTAGAATTCCAGGTGTAGCGGTGAAATGCGTAKAGATCTGGAGGAATACCGGTGGCGAAGGCGGCCCCCTGGACSAAGACTGACGCTCAGGTGCGAAAGCGTGGGGAGCAAACAGGATTAGATACCCTGGTAGTCCACGCCGTAAACGATGTCKACTTGGAGGTTGTGCCCTTGAGGCGTGGCTTCCGGAGCTAACGCGTTAAGTCGCCGCCTGGGGAGTACGGCCGCAAGGTTAAAACTCAAATGAATTGACGGGGGCCCGCACAAGCGGTGGAGCATGGGTTTAATTCGATGCAACGCGAAACCTTACCTGGTCTTGACATCCACGGAAGTTTTCAGAGATGAGAATGTGCCTTCGGGAACCGTGAGACAGGTGCTGCATGGCTGTCTCAGCTCGTGTTGTGAAATGTTGGGTTAAGTCCCGCACGAGCGCAACCCTTATCCTTTGTTGCCAGCGGTCCGGCCGGGAACTCAAAGGAGACTGCCAGTGATAAACTGGAGGAAGGTGGGGATGACGTCAAGTCATCATGGCCCTTACGACCAGGGCTACACACGTGCTACAATGGCGCATACAAAGAGAAGCGACCTCGCGAGAGCAAGCGGACCTCATAAAGTGCGTCGTAGTCCGGATTGGAGTCGCAACTCGACTCCATGAAGTCGGAATCGCTAGTAATCGTGGATCAGAATGCCACGGGAATACGTTCCCGGGCCTGTACACACCCCCGTCACACCATGGGAGTGGGTGCAAAAGAAGTAGTGGTTCGACCTGGCTACGAGAATCCGCGACGCG

**Selected sequence for blast run (39-1386 bp)**

AAGCTTGCTKCTTTGCTGACGAGTGGCGGWCGGGTGAGTAATGTCTGGGAAACTGCCTGATGGAGGGGGATAACTACTGGAAACGGTAGCTAATACCGCATAACGTCGCRRGACCAAAGAGGGGGACCTTCGGGCCTCTTGCCATCGGATGTGCCCAGATGGGATTAGCTAGTAGGTGGGGTAACGGCTCACCTAGGCGACGATCCCTAGCTGGTCTGAGAGGATGACCRGCCACRCTGGAACTGAGACACGGTCCASACTCCTACGGGAGGCAGCAGTGGGGAATATTGCACAATGGGCGCAAGCCTGATGCASCCATGCCGCGKGTATGAAKAAGGCCTTCGGGTTGTAAAGTACTTTCAGCGGGGAGGAAGGGAGTAAAGTTAATACCTTTGCTCATTGACGTTACCCGCASAAKAAGCACCGGCTAACTCCGTGCCAGCAGCCGCGGTAATACGGAGGGTGCAAGCGTTAATCGGAATTACTGGGCGTAAAGCGCACGSAGGCGGTTTGTTAAGTCAGATGTGAAATCCCCGGGCTCAACCTGGGAACTGCATCTGATACTGGCAAGCTTGAGTCTCGTAGAGGGGGGTAGAATTCCAGGTGTAGCGGTGAAATGCGTAKAGATCTGGAGGAATACCGGTGGCGAAGGCGGCCCCCTGGACSAAGACTGACGCTCAGGTGCGAAAGCGTGGGGAGCAAACAGGATTAGATACCCTGGTAGTCCACGCCGTAAACGATGTCKACTTGGAGGTTGTGCCCTTGAGGCGTGGCTTCCGGAGCTAACGCGTTAAGTCGCCGCCTGGGGAGTACGGCCGCAAGGTTAAAACTCAAATGAATTGACGGGGGCCCGCACAAGCGGTGGAGCATGGGTTTAATTCGATGCAACGCGAAACCTTACCTGGTCTTGACATCCACGGAAGTTTTCAGAGATGAGAATGTGCCTTCGGGAACCGTGAGACAGGTGCTGCATGGCTGTCTCAGCTCGTGTTGTGAAATGTTGGGTTAAGTCCCGCACGAGCGCAACCCTTATCCTTTGTTGCCAGCGGTCCGGCCGGGAACTCAAAGGAGACTGCCAGTGATAAACTGGAGGAAGGTGGGGATGACGTCAAGTCATCATGGCCCTTACGACCAGGGCTACACACGTGCTACAATGGCGCATACAAAGAGAAGCGACCTCGCGAGAGCAAGCGGACCTCATAAAGTGCGTCGTAGTCCGGATTGGAGTCGCAACTCGACTCCATGAAGTCGGAATCGCTAGTAATCGTGGATCAGAATGCCACGGGAATACGTTCCCGGGCCTGTACACACCCCCGTCACACCATGGGAGTGGGTGCAAAAGAAGTAG

**Blast result of *E. coli* (lactose positive):**

***Escherichia coli* strain Y4-2 16S ribosomal RNA gene, partial sequence**

**Sequence ID:**[**MT192517.1**](https://www.ncbi.nlm.nih.gov/nucleotide/MT192517.1?report=genbank&log$=nuclalign&blast_rank=1&RID=FUFR7R7U013)**, Length: 1439, Number of Matches: 1**

Range 1: 42 to 1399

Score: 2385 bits (1291), Expect: 0.0, Identities: 1332/1358 (98%), Gaps: 10/1358 (0%), Strand: Plus/Plus

Query 39 AAGCTTGCTKCTTTGCTGACGAGTGGCGGWCGGGTGAGTAATGTCTGGGAAACTGCCTGA 98

||||||||| ||||||||||||||||||| ||||||||||||||||||||||||||||||

Sbjct 42 AAGCTTGCTGCTTTGCTGACGAGTGGCGGACGGGTGAGTAATGTCTGGGAAACTGCCTGA 101

Query 99 TGGAGGGGGATAACTACTGGAAACGGTAGCTAATACCGCATAACGTCGCRRGACCAAAGA 158

||||||||||||||||||||||||||||||||||||||||||||||||| |||||||||

Sbjct 102 TGGAGGGGGATAACTACTGGAAACGGTAGCTAATACCGCATAACGTCGCAAGACCAAAGA 161

Query 159 GGGGGACCTTCGGGCCTCTTGCCATCGGATGTGCCCAGATGGGATTAGCTAGTAGGTGGG 218

||||||||||||||||||||||||||||||||||||||||||||||||||||||||||||

Sbjct 162 GGGGGACCTTCGGGCCTCTTGCCATCGGATGTGCCCAGATGGGATTAGCTAGTAGGTGGG 221

Query 219 GTAACGGCTCACCTAGGCGACGATCCCTAGCTGGTCTGAGAGGATGACCRGCCACRCTGG 278

||||||||||||||||||||||||||||||||||||||||||||||||| ||||| ||||

Sbjct 222 GTAACGGCTCACCTAGGCGACGATCCCTAGCTGGTCTGAGAGGATGACCAGCCACACTGG 281

Query 279 AACTGAGACACGGTCCASACTCCTACGGGAGGCAGCAGTGGGGAATATTGCACAATGGGC 338

||||||||||||||||| ||||||||||||||||||||||||||||||||||||||||||

Sbjct 282 AACTGAGACACGGTCCAGACTCCTACGGGAGGCAGCAGTGGGGAATATTGCACAATGGGC 341

Query 339 GCAAGCCTGATGCASCCATGCCGCGKGTATGAAKAAGGCCTTCGGGTTGTAAAGTACTTT 398

|||||||||||||| |||||||||| ||||||| ||||||||||||||||||||||||||

Sbjct 342 GCAAGCCTGATGCAGCCATGCCGCGTGTATGAAGAAGGCCTTCGGGTTGTAAAGTACTTT 401

Query 399 CAGCGGGGAGGAAGGGAGTAAAGTTAATACCTTTGCTCATTGACGTTACCCGCasaakaa 458

|||||||||||||||||||||||||||||||||||||||||||||||||||||| || ||

Sbjct 402 CAGCGGGGAGGAAGGGAGTAAAGTTAATACCTTTGCTCATTGACGTTACCCGCAGAAGAA 461

Query 459 GCACCGGCTAACTCCGTGCCAGCAGCCGCGGTAATACGGAGGGTGCAAGCGTTAATCGGA 518

||||||||||||||||||||||||||||||||||||||||||||||||||||||||||||

Sbjct 462 GCACCGGCTAACTCCGTGCCAGCAGCCGCGGTAATACGGAGGGTGCAAGCGTTAATCGGA 521

Query 519 ATTACTGGGCGTAAAGCGCACGSAGGCGGTTTGTTAAGTCAGATGTGAAATCCCCGGGCT 578

|||||||||||||||||||||| |||||||||||||||||||||||||||||||||||||

Sbjct 522 ATTACTGGGCGTAAAGCGCACGCAGGCGGTTTGTTAAGTCAGATGTGAAATCCCCGGGCT 581

Query 579 CAACCTGGGAACTGCATCTGATACTGGCAAGCTTGAGTCTCGTAGAGGGGGGTAGAATTC 638

||||||||||||||||||||||||||||||||||||||||||||||||||||||||||||

Sbjct 582 CAACCTGGGAACTGCATCTGATACTGGCAAGCTTGAGTCTCGTAGAGGGGGGTAGAATTC 641

Query 639 CAGGTGTAGCGGTGAAATGCGTAKAGATCTGGAGGAATACCGGTGGCGAAGGCGGCCCCC 698

||||||||||||||||||||||| ||||||||||||||||||||||||||||||||||||

Sbjct 642 CAGGTGTAGCGGTGAAATGCGTAGAGATCTGGAGGAATACCGGTGGCGAAGGCGGCCCCC 701

Query 699 TGGACSAAGACTGACGCTCAGGTGCGAAAGCGTGGGGAGCAAACAGGATTAGATACCCTG 758

||||| ||||||||||||||||||||||||||||||||||||||||||||||||||||||

Sbjct 702 TGGACGAAGACTGACGCTCAGGTGCGAAAGCGTGGGGAGCAAACAGGATTAGATACCCTG 761

Query 759 GTAGTCCACGCCGTAAACGATGTCKACTTGGAGGTTGTGCCCTTGAGGCGTGGCTTCCGG 818

|||||||||||||||||||||||| |||||||||||||||||||||||||||||||||||

Sbjct 762 GTAGTCCACGCCGTAAACGATGTCGACTTGGAGGTTGTGCCCTTGAGGCGTGGCTTCCGG 821

Query 819 AGCTAACGCGTTAAGTCG-CCGCCTGGGGAGTACGGCCGCAAGGTTAAAACTCAAATGAA 877

|||||||||||||||||| |||||||||||||||||||||||||||||||||||||||||

Sbjct 822 AGCTAACGCGTTAAGTCGACCGCCTGGGGAGTACGGCCGCAAGGTTAAAACTCAAATGAA 881

Query 878 TTGACGGGGGCCCGCACAAGCGGTGGAGCATG-GGTTTAATTCGATGCAACGCGA--AAC 934

|||||||||||||||||||||||||||||||| |||||||||||||||||||||| |||

Sbjct 882 TTGACGGGGGCCCGCACAAGCGGTGGAGCATGTGGTTTAATTCGATGCAACGCGAAGAAC 941

Query 935 CTTACCTGGTCTTGACATCCACGGAAGTTTTCAGAGATGAGAATGTGCCTTCGGGAACCG 994

||||||||||||||||||||||||||||||||||||||||||||||||||||||||||||

Sbjct 942 CTTACCTGGTCTTGACATCCACGGAAGTTTTCAGAGATGAGAATGTGCCTTCGGGAACCG 1001

Query 995 TGAGACAGGTGCTGCATGGCTGTC-TCAGCTCGTGTTGTGAAATGTTGGGTTAAGTCCCG 1053

|||||||||||||||||||||||| |||||||||||||||||||||||||||||||||||

Sbjct 1002 TGAGACAGGTGCTGCATGGCTGTCGTCAGCTCGTGTTGTGAAATGTTGGGTTAAGTCCCG 1061

Query 1054 C-ACGAGCGCAACCCTTATCCTTTGTTGCCAGCGGTCCGGCCGGGAACTCAAAGGAGACT 1112

| ||||||||||||||||||||||||||||||||||||||||||||||||||||||||||

Sbjct 1062 CAACGAGCGCAACCCTTATCCTTTGTTGCCAGCGGTCCGGCCGGGAACTCAAAGGAGACT 1121

Query 1113 GCCAGTGATAAACTGGAGGAAGGTGGGGATGACGTCAAGTCATCATGGCCCTTACGACCA 1172

||||||||||||||||||||||||||||||||||||||||||||||||||||||||||||

Sbjct 1122 GCCAGTGATAAACTGGAGGAAGGTGGGGATGACGTCAAGTCATCATGGCCCTTACGACCA 1181

Query 1173 GGGCTACACACGTGCTACAATGGCGCATACAAAGAGAAGCGACCTCGCGAGAGCAAGCGG 1232

||||||||||||||||||||||||||||||||||||||||||||||||||||||||||||

Sbjct 1182 GGGCTACACACGTGCTACAATGGCGCATACAAAGAGAAGCGACCTCGCGAGAGCAAGCGG 1241

Query 1233 ACCTCATAAAGTGCGTCGTAGTCCGGATTGGAGTC-GCAACTCGACTCCATGAAGTCGGA 1291

||||||||||||||||||||||||||||||||||| ||||||||||||||||||||||||

Sbjct 1242 ACCTCATAAAGTGCGTCGTAGTCCGGATTGGAGTCTGCAACTCGACTCCATGAAGTCGGA 1301

Query 1292 ATCGCTAGTAATCGTGGATCAGAATGCCACGG-GAATACGTTCCCGGGCCT-GTACACAC 1349

|||||||||||||||||||||||||||||||| |||||||||||||||||| ||||||||

Sbjct 1302 ATCGCTAGTAATCGTGGATCAGAATGCCACGGTGAATACGTTCCCGGGCCTTGTACACAC 1361

Query 1350 C-CCCGTCACACCATGGGAGTGGGTGCAAAAGAAGTAG 1386

| ||||||||||||||||||||||||||||||||||||

Sbjct 1362 CGCCCGTCACACCATGGGAGTGGGTGCAAAAGAAGTAG 1399
