# Supplementary material for: Multi-drug resistant (MDR) Gram-negative pathogenic bacteria isolated from poultry in the Noakhali region of Bangladesh
Source: PLoS One. 2024 Aug 1;19(8):e0292638. doi: 10.1371/journal.pone.0292638 (PMC11293736; doi:10.1371/journal.pone.0292638)
Supplement: Attachment — Submitted filename: Response to Reviewers.docx [file pone.0292638.s023.docx]

Dear Editor,

Thank you very much for allowing us the opportunity to submit the revised version of our manuscript entitled “Unveiling multi-drug resistant (MDR) gram negative pathogenic bacteria from poultry chickens in the Noakhali region of Bangladesh” for publication in the ‘Plos One'. We really appreciate your effort for precious feedbacks and insightful suggestions for the improvement of our manuscript. We have included all the suggestions as recommended by you. Please see below, in blue text, for a point-by-point response to the editor’s comments and concerns

**#Editor’s comment 1:** Please ensure that your manuscript meets PLOS ONE's style requirements, including those for file naming.

**Authors’ Response:** We express our gratitude to the editor for providing valuable guidance. In response to PLOS ONE's style requirements, we have now rectified the file names accordingly.

**#Editor’s comment 2:** Please state what role the funders took in the study. If the funders had no role, please state: "The funders had no role in study design, data collection and analysis, decision to publish, or preparation of the manuscript."

**Authors’ Response:** The funders contributed solely by providing financial support for this study. They were not involved in the study's design, data collection, analysis, the decision to publish, or the preparation of the manuscript.

**#Editor’s comment 3:** Please remove any funding-related text from the manuscript and let us know how you would like to update your Funding Statement.

**Authors’ Response:** Following the editor's guidance, we have removed the funding-related text from the manuscript. We kindly request that you update the funding statement as follows: 'This work received financial support from the 'Noakhali Science and Technology University-Research Cell' Teachers' grant for the budget year 2021-2022 (Project ID: NSTU/RC-BG-06/T-23/32). We appreciate your attention to this matter.

**#Editor’s comment 4:** We note that you have indicated that data from this study are available upon request. PLOS only allows data to be available upon request if there are legal or ethical restrictions on sharing data publicly. In your revised cover letter, please address the following prompts:

a) If there are ethical or legal restrictions on sharing a de-identified data set, please explain them in detail (e.g., data contain potentially sensitive information, data are owned by a third-party organization, etc.) and who has imposed them (e.g., an ethics committee). Please also provide contact information for a data access committee, ethics committee, or other institutional body to which data requests may be sent.

b) If there are no restrictions, please upload the minimal anonymized data set necessary to replicate your study findings as either Supporting Information files or to a stable, public repository and provide us with the relevant URLs, DOIs, or accession numbers. For a list of acceptable repositories, please see http://journals.plos.org/plosone/s/data-availability#loc-recommended-repositories.

We will update your Data Availability statement on your behalf to reflect the information you provide.

**Authors’ Response:** We express our gratitude to the editor for addressing this crucial matter. There are no ethical or legal restrictions preventing us from sharing a de-identified dataset. Therefore, we are pleased to share our raw dataset as a supplementary file. In the earlier version of the manuscript, a significant portion of the dataset was already included in the supplementary information. In the current version, we are uploading an Excel file (named 'Raw_data') containing the most essential raw data information for this study. We believe that the information provided in the supplementary files is comprehensive enough to replicate the study

**#Editor’s comment 5:** PLOS ONE now requires that authors provide the original uncropped and unadjusted images underlying all blot or gel results reported in a submission’s figures or Supporting Information files. This policy and the journal’s other requirements for blot/gel reporting and figure preparation are described in detail at https://journals.plos.org/plosone/s/figures#loc-blot-and-gel-reporting-requirements and https://journals.plos.org/plosone/s/figures#loc-preparing-figures-from-image-files. When you submit your revised manuscript, please ensure that your figures adhere fully to these guidelines and provide the original underlying images for all blot or gel data reported in your submission. See the following link for instructions on providing the original image data: <https://journals.plos.org/plosone/s/figures#loc-original-images-for-blots-and-gels>.

**Authors’ Response:** In compliance with the PLOS ONE journal guidelines and following the editor's suggestion, we are pleased to share the unaltered gel images from this study. All the uncropped raw images have been consolidated in a single PDF file labeled ‘Raw gel image’

**#Editor’s comment 6:** Please resubmit Figure 1, as the legend is not readable.

**Authors’ Response:** In the initial version of the manuscript, we included a figure with a resolution of 300 dpi, featuring a clearly legible legend. Unfortunately, during the PDF conversion for manuscript submission, the figure resolution was compromised, rendering the legend unreadable. We have now uploaded a new figure, although we cannot guarantee that the image will remain unaffected. In the event of a recurrence, we are willing to provide the image as an email attachment for clarity.

**#Editor’s comment 7:** Ensure that you resubmit Figure 3 because it is not visible.

**Authors’ Response:** Based on the editor's feedback, we have resubmitted Figure 3 with a higher resolution. We are optimistic that this revision will ensure the visibility of the figure in the submitted manuscript file.

**#Editor’s comment 8:** Resubmit a clearer Figure 4A and 4B with legible legends.

**Authors’ Response:** We have resubmitted Figure 4A and 4B with the correction of legible legends mentioned by editor.

**#Editor’s comment 9:** Upload a new Fig. 7 with a legible legend.

**Authors’ Response:** Due to the background color, the legends in Figure 7 were not visible. We have addressed this issue by replacing it with a resubmitted Figure 7 featuring a white background.

**#Editor’s comment 10:** Make sure that the table 2 rows are not breaking across pages (with text in between)

**Authors’ Response:** We express our gratitude to the editor for bringing this unintentional error to our attention. Subsequently, we have rectified Table 2, ensuring that the rows no longer break across the page.

**#Editor’s comment 11:** Ensure that you remove the text highlight color used inside the manuscript.

**Authors’ Response:** We have removed the text highlights from the revised manuscript.
