# Supplementary material for: Multi-drug resistant (MDR) Gram-negative pathogenic bacteria isolated from poultry in the Noakhali region of Bangladesh
Source: PLoS One. 2024 Aug 1;19(8):e0292638. doi: 10.1371/journal.pone.0292638 (PMC11293736; doi:10.1371/journal.pone.0292638)
Supplement: Attachment — Submitted filename: Response to reviewer.docx [file pone.0292638.s024.docx]

Dear Editor,

Thank you very much for allowing us the opportunity to submit the revised version of our manuscript entitled “Unveiling multi-drug resistant (MDR) Gram-negative pathogenic bacteria from poultry chickens in the Noakhali region of Bangladesh” for publication in the ‘Plos One'. We really appreciate the time and that you and the reviewers dedicated to providing feedback on our manuscript and are grateful for the insightful comments on and valuable improvements to our paper. We have incorporated all the suggestions made by the reviewers. Please see below, in blue, for a point-by-point response to the reviewers’ comments and concerns.

**Reviewer #1:**

#Comment 1: Line 1-2. The “Unveiling” in the title sounds a bit inappropriate and therefore suggests that the title reads “Multi-drug resistant (MDR) gram negative pathogenic bacteria isolated from poultry in the Noakhali region of Bangladesh”. Poultry chicken sounds repetitive.

**Author’s response:** We express our gratitude to the reviewer for providing the valuable guidance. In response to reviewer suggestion, we have changed the title in the revised manuscript.

#Comment 2: Line 38-39: “is yet to understand” must read “is yet to be understood”.

**Author’s response:** We have changed in the revised manuscript.

#Comment 3: Line 30-31: I suggest “ESBL”, and other first mentions must be appropriately defined

**Author’s response:** According to the reviewer suggestion we have added the abbreviations in the revised manuscript.

#Comment 4: Line 53: Now-a-days must be replaced with “Nowadays” or better still, “More recently”

**Author’s response:** We have replaced the word in the revised manuscript.

#Comment 5: “Try to rephrase this statement. Perhaps the sentence will read good if " all kinds of people are benefited" is replaced with " many people benefit”.

**Author’s response:** We have rephrased the sentence as per reviewer’s suggestion.

#Comment 6: Consider changing serovars to spp. and for uniformity, stick to spp.

**Author’s response:** In the revised manuscript, we have changed all serovars to spp. according to the reviewer’s advice.

**#Comment 7**: Just for clarification. Is this supposed to be 1ul or 100ul or 1ml as 1µl currently reported seems so small for centrifuging and pipetting off supernatant.

**Author’s response**: We are grateful to the reviewer for identifying this unintentional error. Thanks to their diligence, we have now corrected the amount of broth culture required for DNA extraction in the revised manuscript.

#Comment 8: “S1, S2 and S3 Tables” should be better placed as: “Supplementary data S1, S2 and S3 Tables”

**Author’s response:** We have corrected the data and table information in the revised manuscript.

#Comment 9: Line 195: The referencing of “Hudzicki” must be done well.

**Author’s Response:** We are thankful to the reviewer for his/her prudent suggestions. According to suggestions we have made the changes in the revised manuscript.

Comment 10: The “Selection and criteria of study site” must be incorporated into the methodology and not results as it presents no results.

**Author’s Response**: In response to the reviewer's feedback, we have replaced the 'Selection and Criteria of Study Site' section within the method section of the revised manuscript.

Comment 11: Line 399-400: The sentence does not read right, and rephrasing will do.

**Author’s response:** we have rephrased the sentence as reviewer suggested.

Comment 12: Line 413-414: Fig. 7 is good. However, if a table showing the various measured of the zones of inhibition as against the standards, it would have been great. This link can provide the reference point for number of antimicrobials: https://www.cdc.gov/narms/antibiotics-tested.html.

**Author’s response:** We extend our gratitude to the reviewer for their invaluable advice. Following their guidance, we have included additional supplementary tables (S13) detailing the measured zone of inhibition (ZOI) for each antibiotic. Additionally, we have integrated this ZOI information into Figure 7

Comment 13: Line 422: “Poultry” must be replaced with “Poultry production” to make the sentence read better.

**Author’s response:** We have replaced the word in the revised manuscript.

Comment 14: Line 454: “To our best known” should read “To the best of our knowledge" to sound better.

**Author’s response:** We have made the change in the revised manuscript.

Comment 15: Line 471: “In compare” should read “In comparison.”

**Author’s response:** We have replaced the words as reviewer suggested in the revised manuscript.

**Reviewer #2:**

Comment 1: In title and all the manuscript, write ‘Gram’ not ‘gram’: Unveiling multi-drug resistant (MDR) Gram-negative pathogenic bacteria from poultry chickens in the Noakhali region of Bangladesh.

**Author’s response:** We are grateful to the reviewer for his suggestion. We have corrected the word in the revised manuscript.

Comment 2: Lin 53, write :’... antibiotic resistant (AR) bacteria....’

**Author’s response:** we have added the word as reviewer suggested.

Comment 3: In line 61, you provide the quantity of used antibiotics (8,164,662 kg); however, the reference is since 2009 (Dhanarani TS, Shankar C, Park J, Dexilin M, Kumar RR, Thamaraiselvi K. Study on acquisition of bacterial antibiotic resistance determinants in poultry litter. Poultry science. 2009;88(7):1381-7), this is a very ancient data. Please provide more recent data, now we are in 2024, certainly the quantity of antibiotics used was changed.

**Author’s response:** We have added the recent data with citation as reviewer suggested in the revised manuscript.

Comment 4: The term ‘Enterobacteriaceae’ must be written in italic, verif this in all the manuscript.

**Author’s response:** We have ensured that all instances of the term '*Enterobacteriaceae*' are correctly formatted in italics throughout the revised manuscript

Comment 5: Correct as follow: ‘Enterobacteriaceae frequently serve as carriers of extended-spectrum β-lactamase (ESBL)-encoding genes and are frequently........’

**Author’s response:** We are thankful to the reviewer for the suggestion and corrected in the revised manuscript.

Comment 6: Correct as follows: ‘..The presence of blaCTX-M, blaSHV, and blaTEM genes encoding CTX-M, TEM, and SHV β-lactamases, respectively, empowers these bacteria with resistance against penicillins, first, second, and third-generation cephalosporins, as well as aztreonam (17, 18).’ PLEASE ‘bla’ must be in italic and the name of the enzyme as indice (under ‘bla’) and not in italic.

**Author’s response:** We extend our gratitude to the reviewer for their invaluable feedback. As per the reviewer's advice, we have implemented the necessary corrections.

Comment 7: In the sentence ‘Notably, studies by Kluytmans et al. and Leverstein van Hall et al. have highlighted strong genetic similarities between ESBL-producing E. coli 90 isolated from chicken meat and humans’ Please add the number of the references ‘Kluytmans et al. (its number in the list of reference)’ and ‘Leverstein van Hall et al (its number in the list of reference).

**Author’s response:** We are grateful to the reviewer for identifying our unintentional error. We have promptly rectified this oversight by adding the reference to the list

Comment 8: Line 163, in DNA extraction, I believe that you centrifuged 1 ml of culture not just 1 µl; please verify and correct this. IN ADDITION, just a remark, 30 min of heating is a lot, 10min to 12 min at 100 °C is enough to get the lyses of bacteria, especially Gram-negative bacteria with fragile peptidoglycan wall.

**Author’s response:** We appreciate the reviewer for identifying our typo mistake. We have corrected the measurement from 1 µl culture to 1 ml of culture. Additionally, we thank reviewer for his insightful remarks. Going forward, we will explore reduced heating times for DNA extraction from Gram-negative bacteria

Comment 9: DNA extraction, please delete the step ‘x’; you already collected supernatant that contain DNA for PCR, why you heat it again??? This is incorrect, in this step ‘x’ you will make DNA in two strands, why do it?, this step can degrade DNA, which false PCR.

**Author’s response:** In accordance with the reviewer's suggestion, we have removed step 'x' from the DNA extraction protocol. Employing this revised protocol, we successfully extracted DNA from Gram-negative bacteria for another project. We appreciate the valuable advice, which not only streamlined our protocol but also reduced the DNA extraction time. Thank you for your guidance.

Comment 10: Line 195, please correct it is ‘2009’ not ‘20090’ (.....Kirby-Bauer disk-diffusion method according to Hudzicki, (20090 (43).’ I do not know if life still to that dates 20090.

**Author’s response:** We have now corrected the typo. Thank you for bringing it to our attention.

Comment 11: In antimicrobial susceptibility test you divided beta-lactams to : ‘Ampicillin (AMP25) and Amoxicillin-clavulanic acid (AMC 30) from β-lactams ; Cefotaxime 201 (CTX 30) and Cefoxitin (CX 30) from Cephems; Aztreonam (AT 30) from monobactams; Imipenem (IMP 10) from carbapenems. THIS classification is somewhat correct. However, these antibiotics must be considered as one group (Beta-lactams) when you define MDR. The MDR is for strains showing resistance to three or more families (Beta-lactams are: Ampicillin, Amoxicillin-clavulanic acid; Cefotaxime, Cefoxitin; Aztreonam; Imipenem), so be careful when defining MDR strains.

**Author’s response:** We express our gratitude to the reviewer for their insightful feedback. We have diligently incorporated the suggested correction into the revised manuscript.

Comment 12: Line 226, tou wrote ‘...alongside the New Delhi Metallo β-lactamase (blaNDM) gene’. PLEASE INDICATE THAT IT CODE A metallo-carbapenemase to avoid misunderstanding as an ESBL gene; NDM is not an ESBL.

**Author’s response:** We have revised the sentence based on the reviewer's advice

Comment 13: Line 229, you wrote ‘...while sul1 and sul2 were examined for Trimethoprim-Sulfamethoxazole resistance’. In reality this is not perfectly correct. The sul 1, sul 2 and sul3 encode resistance to sulfonamides not to trimethoprim. Yes when the strain is resistant to sulfamethoxazole it can be also resistant to trimethoprim since the biochemical process of folates synthesis is interrupted. Trimethoprime-resistance is encoded by dhfr genes.

**Author’s response:** We sincerely appreciate the invaluable advice provided by the reviewer. Following their guidance, we have now rectified the sentence accordingly in the revised manuscript

Comment 14: Line 230, modify the sentence as follow:’Moreover, among the globally described ten mcr variants encoding colistin resistance, only mcr-1 gene was investigated by PCR since it is more prevalent worldwide.’

**Author’s response:** We extend our gratitude to reviewer for improving the sentence.

Comment 15: Figure 1 (A and B), please correct ‘K. pneumoniae’ not ‘K. pneumonia’. Correct this also in Figure 2 and Table 1 and table 2, verify this in all the manuscript. Please correct ‘gyr B’ not ‘gyr-b’ in figure 2.

**Author’s response:** We have corrected the following suggestions made by reviewer in the revised manuscript.

Comment 16: PLEASE IN THE TEXT PROVIDE THE NUMBER OF ISOLATES OF EACH IDENTIFIED SPECIES. I want to know the number of E. coli, K. pneumoniae...... Please provide the number of species and the percentages (from broiler and from layer; and from the total). For example, after reading the article I do not know the number of E. coli isolates!!!!.

**Author’s response:** We have added the number of isolates of each identified species in the revised manuscript.

Comment 17: WHEN you provide any data about antimicrobial resistance or genes please provide the number and the frequency (n (%)).

**Author’s response**: With all due respect to the reviewer, we would like to offer clarification regarding why we did not present the number and frequency of analyzed antimicrobial genes. In our antibiogram test, we meticulously displayed the comprehensive antibiotic resistance profiles of all isolates for each species (refer to Table 2). However, for the purpose of DNA extraction and MDR gene detection via PCR, we deliberately selected only the top three isolates from each species based on their highest MAR index. Our intention was to establish a correlation between the phenotypic antibiotic resistance results and the PCR-based detection of antibiotic-resistant genes. Hence, the omission of the specific number and frequency of antibiotic-resistant genes was motivated by our focus on this correlation

Comment 18: Line 289, write: ‘Phylogenetic analysis of identified bacterial isolates’

**Author’s response:** We have changed this according to the reviewer suggestion.

Comment 19: In section ‘results’, ‘Phenotypic characterization of antibiotic resistant pattern’ need revision. Please try to present percentages of resistance in simple form. Just few sentences can explain the detected resistance. The paragraph is very long, try to shorten it by presenting simple and clear results. Readers cannot follow what you wrote. PLEASE COMAPARISON OR CONCLUSION OR DISCUSSION OF YOUR RESULTS MUST BE DONE IN SECTION ‘DISCUSSION’

**Author’s response:** We extend our gratitude to the reviewer for their invaluable advice. We concur with the reviewer's observation regarding the size of this section. In response to previous suggestions, such as incorporating isolate numbers and percentages, we have made essential revisions. Additionally, in line with the reviewer's guidance, we have endeavored to streamline this section, focusing solely on the analysis findings. We anticipate that these revisions will ease readers to follow the story of the study.

Comment 20: In section Results, sentences should be short, clear; especially you provided a lot of tables, figures and supplementary files, sometimes are enough. Paragraphs are in bloc, try to subdivide them. Readers cannot read paragraph containing 20 lines, when you cut the long paragraph to 3 paragraphs it will be better

**Author’s response:** In response to the reviewer's advice and valuable feedback, we have implemented necessary corrections in the results section. While ensuring the original narrative remains intact, we have removed several sentences, resulting in a slightly shorter revised section compared to the previous version.

Comment 21: Change the title of table 3, as follows: Table 3: Antimicrobial susceptibilities of Gram-negative isolates collected from broiler and layer chicken samples.

**Author’s response:** We have removed the table 3 according to reviewer suggestion.

Comment 22: In table 3, I believe that it is not correct to make the sum of all Gram negative bacteria. In your collection there are some species that are naturally resistant to some antibiotics. I advise to delete this table since in table 2 you perfectly provided the resistance profile of each isolate of each species, this is enough. Really I disliked this table 3.

**Author’s response:** We have removed the table 3 according to reviewer suggestion.

Comment 23: For example this paragraph can be as follows (I make few modification also):

Upon evaluating ESBL gene presence and other genes encoding resistance markers in collected isolates, we observed that lactose-positive and lactose-negative E. coli carried blaTEM, tet A, tet B, sul 1, and sul 2 genes, with the latter also harboring blaSHV (Figs 5A, 5B, 5C). These E. coli isolates displayed resistance to β-lactam combination agents, penicillin, cephalosporins, tetracycline, and folate pathway antagonists. Similarly, K. pneumoniae isolates from broiler and layer chickens showed the presence of blaTEM, blaSHV, sul 1, and tet A genes (Fig 5D).

A significant portion of K. pneumoniae isolates from both groups exhibited resistance to β-lactam combination agents, penicillin, cephalosporins, tetracycline, and folate pathway antagonists. Among the isolates, E. hormaechei, P. penneri, P. stuartii, W. chitiniclastica, and M. morganii carried tet A, sul 1, blaSHV, and blaTEM genes (Figs 5E-G, 5I-J) aligning with their phenotypic resistance.

Lastly, S. enterica isolates carried sul 1, sul 2, blaTEM genes (Fig 5H) and most of the S. enterica isolates shown resistance to β-lactam combination agents, penicillin, cephalosporins, tetracycline, and folate pathway antagonists. As those isolates showed resistance to tetracycline without the presence of both tet A and tet B genes indicated that the presence of other genes encoding tetracycline resistance, which were not investigated in this study, in S. enterica isolates.’

**Author’s response:** We are grateful to the reviewer for this suggestion. We have replaced the paragraphs as reviewer suggested in the revised manuscript.

Comment 24: It is not correct to say about genes ‘isoforms’ or ‘isotype’, in biochemistry this is correct but in genetic (gene) this is not correct. You wrote in line 397 ‘mcr1isotype’, this can be wrote in this case as ‘mcr-1 variant’.

**Author’s response:** We have made the change as reviewer suggested.

Comment 25: Please verify the order of figures and tables.

**Author’s response:** According to the reviewer suggestion we have verified the order of the figures and tables.

Comment 26: The mcr1 must be written in all the text and figures and tables as ‘mcr-1’, this is the international nomenclature of mcr genes.

**Author’s response:** We are appreciative to the reviewer suggestion and change the correctd mcr gene name in the revised manuscript.

Comment 27: Discussion is very long try to shorten it.

**Author’s response**: In accordance with the reviewer's suggestion, we have condensed the discussion section while preserving the original meaning of the revised manuscript

Comment 28: PLEASE IN SECTION DISCUSSION NEVER REFER TO TABLES AND FIGURES, THIS WAS ALREADY DONE IN SECTION RESULTS.

**Author’s response:** We are thankful to reviewer. According to the reviewer suggestion we have removed the table and figure citations from discussion.
