# Supplementary material for: Multi-drug resistant (MDR) Gram-negative pathogenic bacteria isolated from poultry in the Noakhali region of Bangladesh
Source: PLoS One. 2024 Aug 1;19(8):e0292638. doi: 10.1371/journal.pone.0292638 (PMC11293736; doi:10.1371/journal.pone.0292638)
Supplement: Attachment — Submitted filename: Response to reviewer_R3.docx [file pone.0292638.s025.docx]

Dear Editor,

Thank you very much for allowing us the opportunity to submit the revised version of our manuscript entitled “Multi-drug resistant (MDR) Gram-negative pathogenic bacteria isolated from poultry in the Noakhali region of Bangladesh” for publication in the ‘Plos One'. We really appreciate the time and that you and the reviewers dedicated to providing feedback on our manuscript and are grateful for the insightful comments on and valuable improvements to our paper. We have incorporated all the suggestions made by the reviewers and you. Please see below, in blue, for a point-by-point response to the reviewers’ and your comments and concerns.

**#Editor**

**#Editor’s comment 1:** Please resubmit Figure 1, as the legend is not readable.

**Author’s response:** In the initial version of the manuscript, we included a figure with a resolution of 300 dpi and a clearly legible legend. Unfortunately, during the PDF conversion for manuscript submission, the figure resolution was compromised, making the legend unreadable. We have now uploaded a new figure. Additionally, we have attached Figure 1 to the email in case the image quality is compromised in the online version.

**#Editor’s comment 2:** Ensure that you resubmit Figure 3 because it is not visible.

**Author’s response:** Based on the editor's feedback, we have resubmitted Figure 3 with a higher resolution. We are optimistic that this revision will ensure the visibility of the figure in the submitted manuscript file. Additionally, we have submitted the Figure 3 in the E-mail attachment.

**#Editor’s comment 3:** Resubmit a clearer Figure 4A and 4B with legible legends.

**Author’s response:** We have resubmitted Figure 4A and 4B with the correction of legible legends mentioned by editor. Additionally, we have submitted the Figure 4A and 4B in the E-mail attachment

**#Editor’s comment 4:** Upload a new Fig. 7 with a legible legend.

**Author’s response:** We have uploaded a new Figure 7 according to the editor’s advice. Additionally, we have attached Figure 7 to the email in case the image quality is compromised in the online version.

**#Editor’s comment 5:** Make sure that the table 2 rows are not breaking across pages (with text in between)

**Author’s response:** We have rectified Table 2, ensuring that the rows no longer break across the page.

**#Editor’s comment 6:** Ensure that you remove the text highlight color used inside the manuscript.

**Author’s response:** We have thoroughly checked and ensured that there is no text highlight in the revised manuscript

**Reviewer #1:**

The authors should be more diligent in their write up and be more coherent as the whole article is riffed with grammatical errors.

**Author’s response:** We appreciate the reviewer’s valuable feedback. We have thoroughly checked the manuscript for grammatical errors with the help of a native English speaker and identified some errors, which have been corrected in the revised version. Additionally, some sentences have been improved, and these changes can be easily seen in the track change version of the revised manuscript.

**#Comment 1:** Line 1: The title will read better if written as “Multi-drug resistant (MDR) Gram-negative pathogenic bacteria isolated from poultry in the Noakhali region of Bangladesh”

**Author’s response:** We express our gratitude to the Reviewer for his valuable feedback. According to the reviewer’s suggestion we have changed the title in the revised manuscript.

#Comment 2: Line 22: Please change all multi drug to “multi-drug”

**Author’s response:** In the revised manuscript we have changed all the ‘multi drug’ to ‘multi-drug’.

**#Comment 3:** Line 30: “Targeted gene sequences were amplified for detection ….” Should read “Targeted gene sequences were amplified for the detection….

**Author’s response:** We thanks reviewer for his guidance. We have made the necessary change according to reviewer advice.

**#Comment 4:** Line 150: What do authors mean by “distinct chickens”?

**Author’s response:** In the revised manuscript, we have eliminated the word "distinct" from the line.

#Comment 5: Line 159: Authors should state the quantity of Peptone water broth used and better still if the methodology was adopted it must be referenced unless otherwise

**Author’s response:** In accordance with the reviewer's suggestion, we have added the quantity of peptone water broth used for the culture, along with the appropriate citation

#Comment 6: Line 163: Full meaning of XLD is “Xylose Lysine Deoxycholate” and not what the authors have stated.

**Author’s response:** We appreciate the reviewer for identifying our typographical error. We have corrected the full meaning of XLD in the revised manuscript.

#Comment 7: Line 173: The step iii as defined by the authors is unclear. Does this imply that after removal of the supernatant, the same tube is again filled with 1ml of pre-enrichment culture and centrifuged again?

**Author’s response:** We appreciate the reviewer’s query. Yes, after removing the supernatant, the same tube is refilled with 1 ml of pre-enrichment culture and centrifuged again to achieve a higher concentration of bacterial cells.

#Comment 8: Line 193: There is an excess single space after “blastn”

**Author’s response:** We thanks reviewer for bringing this unintentional error to our attention. We remove the excess single space in the revised manuscript.

#Comment 9: Line 199: Authors must provide the online link to this MAFFT tool and appropriately provide a citation

**Author’s response:** We appreciate the reviewer's valuable advice. Following his suggestion, we have included an online link to the MAFFT tool for phylogenetic tree construction, along with the appropriate citation.

#Comment 9: Line 199: Authors must be sure MAFFT was used for building phylogenies as the tool is mostly used for building sequence alignments from which phylogenies are built using tools like ClustaW

**Author’s response:** We have ensured that the MAFFT tool was used for building the phylogenetic tree. Additionally, we have cited relevant articles (references 43 and 44) for MAFFT-based phylogenetic analysis.

#Comment 10: Lines 209-212: There are a series of “;;” which are inappropriate

**Author’s response:** We thank the reviewer for identifying the typos. We have removed the extra semicolon in the revised manuscript.

#Comment 11: Line 285: There is a repetition of “isolates”

**Author’s response**: The redundant word “isolates” has been removed in the revised manuscript.
